# Preconception and Prenatal Nutrition and Neurodevelopmental Disorders: A Systematic Review and Meta-Analysis

**DOI:** 10.3390/nu11071628

**Published:** 2019-07-17

**Authors:** Mengying Li, Ellen Francis, Stefanie N. Hinkle, Aparna S. Ajjarapu, Cuilin Zhang

**Affiliations:** Division of Intramural Population Health Research, *Eunice Kennedy Shriver* National Institute of Child Health and Human Development, National Institutes of Health, Bethesda, MD 20817, USA

**Keywords:** pregnancy nutrition, neurodevelopmental disorders, autism spectrum disorder, attention deficit disorder with hyperactivity, developmental origins of health and disease, systematic review, meta-analysis

## Abstract

Preconception and prenatal nutrition is critical for fetal brain development. However, its associations with offspring neurodevelopmental disorders are not well understood. This study aims to systematically review the associations of preconception and prenatal nutrition with offspring risk of neurodevelopmental disorders. We searched the PubMed and Embase for articles published through March 2019. Nutritional exposures included nutrient intake or status, food intake, or dietary patterns. Neurodevelopmental outcomes included autism spectrum disorders (ASD), attention deficit disorder-hyperactivity (ADHD) and intellectual disabilities. A total of 2169 articles were screened, and 20 articles on ASD and 17 on ADHD were eventually reviewed. We found an overall inverse association between maternal folic acid or multivitamin supplementation and children’s risk of ASD; a meta-analysis including six prospective cohort studies estimated an RR of ASD of 0.64 (95% CI: 0.46, 0.90). Data on associations of other dietary factors and ASD, ADHD and related outcomes were inconclusive and warrant future investigation. Future studies should integrate comprehensive and more objective methods to quantify the nutritional exposures and explore alternative study design such as Mendelian randomization to evaluate potential causal effects.

## 1. Introduction

Maternal nutrition is critical for fetal brain development. Maternal diet prior to pregnancy is important for optimizing nutritional status which plays a vital role in maintaining a healthy pregnancy and supporting the developing fetus [1]. Nutrition around the time of conception is important for gamete function and placental development [2]. Starting 2–3 weeks after fertilization, the embryo undergoes orchestrated processes of neuronal proliferation and migration, synapse formation, myelination, and apoptosis to develop the fetal brain [3]. In this period of rapid development, the brain has heightened sensitivity to the environment, where perturbation may predispose the fetus to postnatal neurodevelopmental disorders [4,5]. Overall, supply of nutrients during the preconception and prenatal periods not only provides the basic building blocks for the brain [6], but may also “program” the brain through epigenetic mechanisms to confer risk or resilience to neurological conditions later in life [7]. The modifiable nature of maternal nutrition during sensitive periods potentially offers opportunities for intervention.

Several nutrients have previously been identified to have a critical role in prenatal neurodevelopment. For example, folate is an essential co-factor in one-carbon metabolism responsible for DNA and RNA synthesis and DNA methylation—processes that are particularly important during periods of rapid growth and development. Inadequate folate intake has been linked to altered DNA methylation [8,9] and compromised fetal brain development [10,11], particularly in animal studies. Preconception and early pregnancy supplementation of folic acid—the synthetic form of folate—was found effective in preventing neural tube defects [12]. In addition to folate, polyunsaturated fatty acids (PUFAs), particularly arachidonic acid (AA) of the omega-6 (n-6) family and docosahexaenoic acid (DHA) of the omega-3 (n-3) family, are structural components of neuronal membrane phospholipids and the myelin sheath insulating the neuronal axons [13]. PUFAs accumulate rapidly in the brain starting in the third trimester and continuing into early postnatal life [14], and the membrane PUFA composition is mainly dependent on maternal dietary supply of DHA [15]. Restriction of DHA and its precursors in the perinatal period negatively impact cognitive and behavioral outcomes in animal studies [16,17].

Some minerals are also known to play important roles in prenatal neurodevelopment. For example, iron is essential for the regulation of neuronal energy metabolism during development; deficiency affects the structure and function of fetal hippocampus, compromising learning and memory [18]. Similarly, iodine is necessary to produce thyroid hormones which regulate brain growth and development; deficiency during the prenatal period results in cognitive deficit [19]. While not a nutrient, caffeine, a psychoactive substance widely consumed through coffee and tea, improves alertness by blocking adenosine receptors in the brain. Prenatal and early postnatal exposure to caffeine alters brain neurochemistry and behavior in animal studies [20]. In addition to individual nutrients and substance, foods and dietary patterns may capture the combined and interactive effects of nutrients in prenatal neurodevelopment.

**Figure 1:**
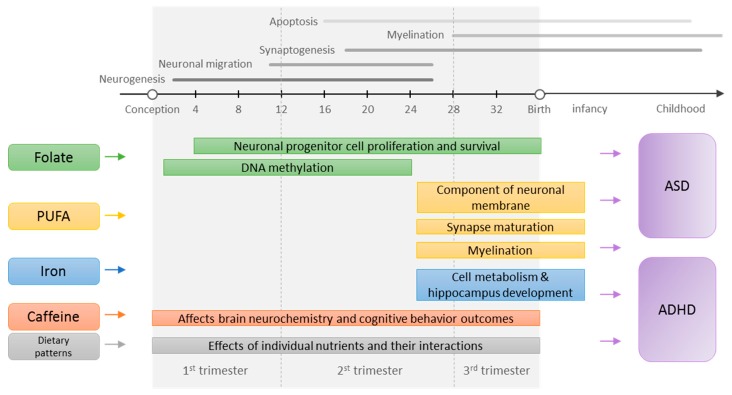
Proposed mechanisms in which preconception and prenatal nutrition affects the risk of autism spectrum disorder (ASD) and attention-deficit/hyperactivity disorder (ADHD). The neurodevelopmental timelines were adapted from the previous publications [3,28]. PUFA, polyunsaturated fatty acids.

In the past decade, accumulating evidence from human epidemiological studies support the link between maternal nutrition and offspring cognitive and behavioral abilities [21,22,23,24,25]. Yet, it is unclear whether maternal nutrition is relevant to the risk of clinically defined neurodevelopmental disorders, which are characterized by deficits that produce impairments in daily functioning. A comprehensive and critical review of the evidence may shed light on the question. Limited studies previously reviewed evidence on maternal folic acid intake and offspring autism spectrum disorder (ASD) risk [26,27]; however, an updated review with a comprehensive critique of the evidence is needed. Furthermore, much of the evidence on maternal nutrition and offspring attention-deficit/hyperactivity disorder (ADHD) risk has not been reviewed. Lastly, critical questions regarding the most sensitive time windows (i.e., before vs. during pregnancy) for nutritional exposures remain unanswered. To address these critical knowledge gaps, we conducted a systematic review of epidemiologic studies examining the association of maternal nutrition before and during pregnancy with the risk of offspring neurodevelopmental disorders; we focused on dietary factors including supplements to inform potential public health interventions specific to maternal diet. To provide quantitative syntheses of evidence, we also performed meta-analyses whenever the data were available. A summary of proposed mechanisms in which preconception and prenatal nutrition affects risks of ASD and ADHD and relevant sensitive windows are presented in a schematic figure (Figure 1).

## 2. Materials and Methods

### 2.1. Eligibility Criteria

The systematic review included studies investigating associations of nutrition before or during pregnancy with offspring major neurodevelopmental disorders, including ASD, ADHD, and intellectual disability (ID). Maternal nutrition was evaluated at multiple levels: nutrient intake, food intake, or dietary patterns. Clinical diagnosis, a positive screening outcome, or a continuous measure of the traits or symptoms of the disorder were considered as outcomes. The studies were conducted in human populations, of case-control, cohort, or experimental design, and published in the English language. Studies were excluded if the exposures were not reflective of maternal dietary intake, such as teratogens (e.g., alcohol), chemicals, certain circulating biomarkers that are not directly reflective of maternal intake (e.g., 25-hydroxyvitamin D and ferritin), biomarkers in umbilical cord blood, or dietary counseling without quantification of actual maternal diet. We also excluded studies where outcomes were not reflective of neurodevelopmental disorders (continuous measures of Intelligence quotient (IQ), or suboptimal IQ defined by a non-clinical cutoff), outcomes were secondary to structural birth defects (e.g., neural tube defect) or chromosomal disorders (e.g., Down Syndrome), relevant associations were not reported, or full-texts were not available. Other neurodevelopmental disorders (i.e., communication disorders, motor disorders, and specific learning disorders) were not covered in this review, as a pilot search found very few epidemiologic studies investigating these disorders in association with maternal nutrition.

### 2.2. Literature Search, Screening and Abstraction

We searched PubMed and Embase on 27 March 2019. Search strategies were developed a priori for each database with assistance from a librarian. The search targeted title, abstract, and subject headings. Search terms consisted of neurodevelopmental disorders (either the general term or specific terms for ASD, ID, or ADHD), nutrition (nutrients, foods, or dietary patterns), prenatal or preconception periods, and observational or experimental design, combined using the Boolean Operator “AND”; additional search terms were used to exclude animal studies. The search strategy and search history are presented in Table S1.

After the systematic search, we screened the title and abstract of the records for eligibility, and then assessed the full-text of the retained records for final inclusion decisions. We also examined the bibliographies of existing literature reviews on maternal nutrition and neurodevelopmental disorders to identify additional articles not covered in the systematic search. We abstracted relevant data in the included articles using a standardized form.

### 2.3. Meta-Analysis

We conducted meta-analyses of associations between specific nutritional exposures and neurodevelopmental outcomes whenever three or more relevant independent estimates were available. The analysis pooled relative risks (RRs) and odds ratios (ORs) estimated from cohort studies; OR is a reasonable approximation of RR in these studies as both ASD (prevalence 1–5%) [29] and ADHD (prevalence 5–10%) [30] are relatively rare. Estimates from case-control studies were not pooled with those from cohort studies due to differences in study design and retrospective recall of the nutritional exposures in these studies (all the case-control studies are retrospective); sensitivity analysis including estimates from the case-control studies did not change the findings substantively. Estimates of hazard ratio were not pooled with the RRs, as it is a conceptually different measure [31]. Pooled effect sizes were estimated using DerSimonian and Laird random-effects model. Studies reporting the diagnosis and the screening outcomes of neurodevelopmental disorders were meta-analyzed separately. Attempts were made to separate the effects of specific nutrients from multivitamins, and between exposures at different time periods (e.g., before pregnancy and during pregnancy), whenever possible. The meta-analysis was performed in Stata version 14 (StataCorp LLC, College Station, TX, USA) [32].

### 2.4. Quality Assessment

While generic tools to evaluate the quality of observational studies exists, these tools may not sufficiently address the methodological concerns important for the topic of interest and the type of studies included. As such, we critiqued the methodological quality of the included studies throughout the narrative review whenever relevant. We also discussed common issues across exposures and outcomes in a separate section following the reviews, with an emphasis for future directions.

## 3. Results and Commentary

**Figure 2:**
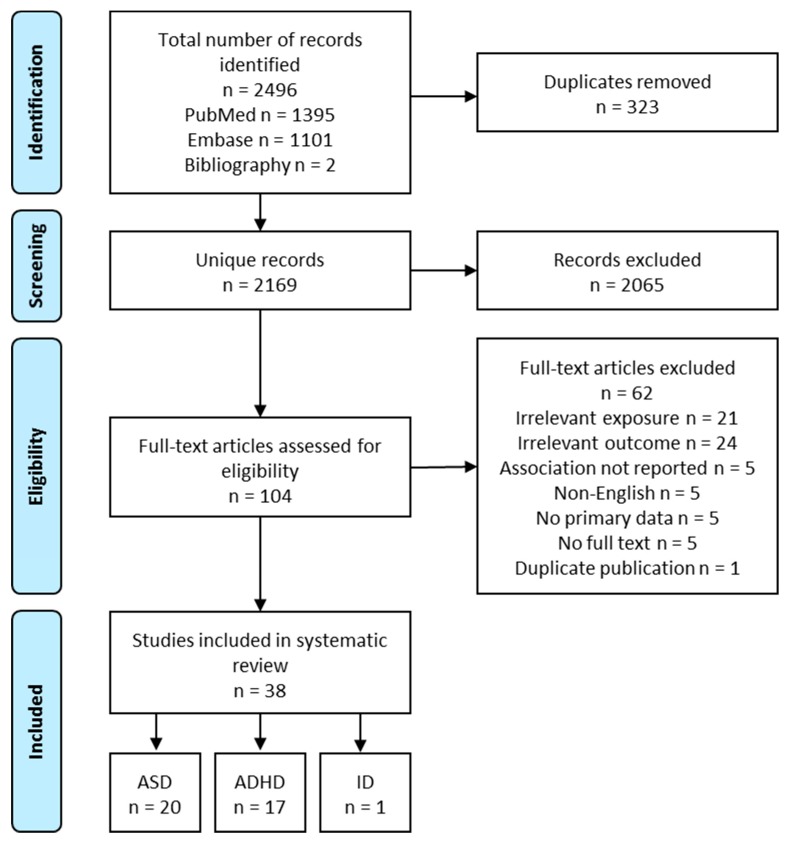
Flow diagram of study selection process, intellectual disability (ID).

A total of 2167 unique records were obtained from the systematic literature search. After screening the titles and abstracts, 102 relevant records remained. After the full-text review, 36 articles were included. Two additional articles were identified from the bibliographies of existing literature reviews, resulting in a total of 38 studies. Overall, 20 studies reported outcomes related to ASD, 17 ADHD, and 1 ID (Figure 2). The following review focuses on ASD and ADHD. Exposures in these studies included vitamins and minerals (i.e., folate, iron, calcium, and iodine), fatty acids (i.e., PUFA), foods (i.e., fish, fruits), supplements (i.e., multivitamins), and dietary patterns. Nutrients intake or status were grouped together with relevant food sources (e.g., folate with multivitamins, PUFA with seafood) to facilitate the interpretation of the findings. Nutrients or foods reported only in a single study were not reviewed below.

### 3.1. Maternal Nutrition and ASD Risk

The characteristics and main findings of the studies on the associations between maternal nutrition and ASD are presented in Table 1. Of the 20 studies, 14 are prospective study and 6 are retrospective studies. These studies were conducted in the USA, Denmark, Norway, Sweden, The Netherlands, Spain, Israel, and China.

#### 3.1.1. Maternal Folate Intake/Status, Multivitamin Intake and Offspring ASD Risk

Fourteen studies examined the association of folic acid supplementation, folate status, or multivitamin intake in relation to offspring ASD risk. Among them, ten are prospective cohort studies [33,34,36,37,38,39,40,41,42], and four are retrospective case-control studies [35,43,45,53]. Studies on folate and multivitamins were combined as it is difficult to separate the two exposures. Among the 12 independent studies [33,34,35,36,37,38,40,41,42,43,53] including 594,229 children (8851 ASD cases), nine found a significant inverse association between maternal folic acid or multivitamin intake during pregnancy (with or without pre-pregnancy use) and offspring ASD risk [33,34,35,37,38,40,41,42,44,45,46], one a null association [37], one a significant positive association [43], and one a curve-linear association with modest intake/status associated with lowest ASD risk [36].

Of the nine studies reporting an inverse association between maternal folic acid or multivitamin intake/status during pregnancy and offspring ASD risk [33,34,35,37,38,40,41,42,45], four were large prospective cohort studies which identified ASD cases through population-based patient registries [38,41,42] or medical records of large health care organizations [34]; the ASD cases were either confirmed by specialist providers [34,41] or independently validated [38,42]. These studies included very large sample sizes (45,000–270,000 participants) and contributed the greatest weight to the overall evidence. Three were smaller cohort studies which assessed ASD risk within the study using validated instruments for ASD diagnosis [33] or screening [40,46]. Two were retrospective case-control studies where ASD cases were identified from service registries [35,44].

There are two common methodological concerns about these studies. First, there is potential measurement error in folic acid or multivitamin intake, which were generally assessed by self-report [33,35,42] or administrative records [34,38,41]. Of note, two studies with additional data on whole blood/plasma folate levels found the self-reported measure, but not the biomarker measure, to be associated with lower ASD risk [40,46]. The inconsistent findings may be due to measurement error in either measure, or differences in measurement timing. Second, there is potential bias due to residual confounding. Women who took folic acid supplements generally had higher socioeconomic status, healthier lifestyle, and lower BMI [33,37,38,42]; some of these factors, such as a lower BMI, may contribute to lower risk of ASD [54]. These factors were not always adjusted for, making the results subject to residual confounding.

Despite the methodological concerns, additional data support an inverse association between maternal folate status and ASD risk. For example, the *methylenetetrahydrofolate reductase* (*MTHFR*) gene encodes the methylenetetrahydrofolate reductase, a rate-limiting enzyme responsible for converting folate to its bioactive form for methylation reactions [55]; the minor T allele at loci *MTHFR* 677 is known to confer higher susceptibility to inadequate folate status [55]. In one study, maternal folic acid supplementation was associated with a much greater reduction of children’s ASD risk when the mother and/or the child had at least one T allele, but the association was null when neither had a T allele [44]. Furthermore, two studies found maternal folic acid supplementation to be specifically associated with a lower risk of ASD, but not associated with the risk of developmental disorders other than ASD [33,44]. If maternal folic acid supplementation was spuriously associated with ASD due to residual confounding, it is likely that it would also show an association with other developmental disorders. Similarly, one study also found the risk of ASD to be specifically associated with folic acid intake, but not fish oil intake [42].

In contrast to the overall finding of an inverse association between folic acid or multivitamin intake/status and ASD risk, a study among 92,676 children in the Danish National Birth Cohort (DNBC) did not find a significant association between folic acid supplementation and ASD risk [37]. Possible explanations include better folate status without supplementation, lower frequency of T alleles at *MTHFR* 677, and insufficient doses of folic acid in the supplements [37,39]. In addition, a study including 1257 children in the Boston Birth Cohort found both low (<2 times/week) and high (>5 times/week) frequencies of multivitamin intake were associated with an elevated risk of ASD, compared to moderate intake (3–5 times/week) [36]. The increased risk associated with high frequencies of folic acid intake may be a result of the above normal level of folate in the study sample [36].

**Figure 3:**
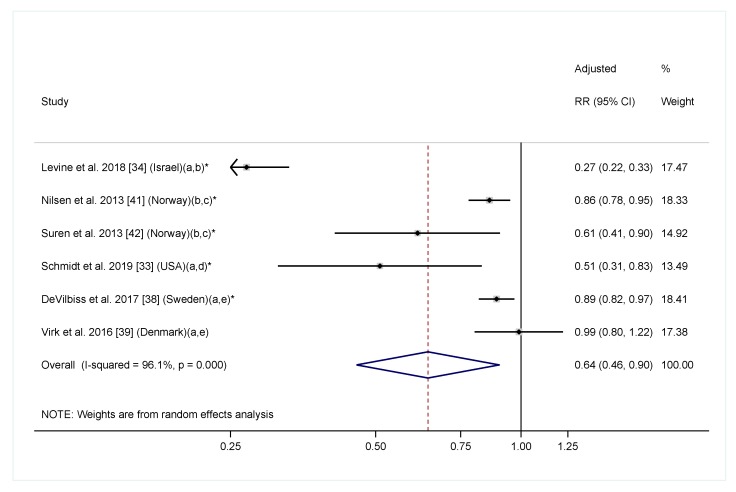
Adjusted relative risk (RRs) of offspring ASD risk associated with maternal intake of supplement containing folic acid, multivitamin or prenatal vitamin during pregnancy (with or without pre-pregnancy use). The overall effect size was estimated using random effects models weighted by inverse variance of each study. One data point was included for each study. Estimates covering any period during pregnancy were included. When estimates for folic acid and multivitamin were both available, the one for folic acid were selected. Notes: (a) Exposure during pregnancy; (b) Intake of supplement containing folic acid vs. no; (c) Exposure before and during pregnancy; (d) Folic acid intake ≥600 mcg/day vs. <600 mcg/day; (e) Intake of multivitamin vs. never/rarely. * *p* < 0.05.

**Figure 4:**
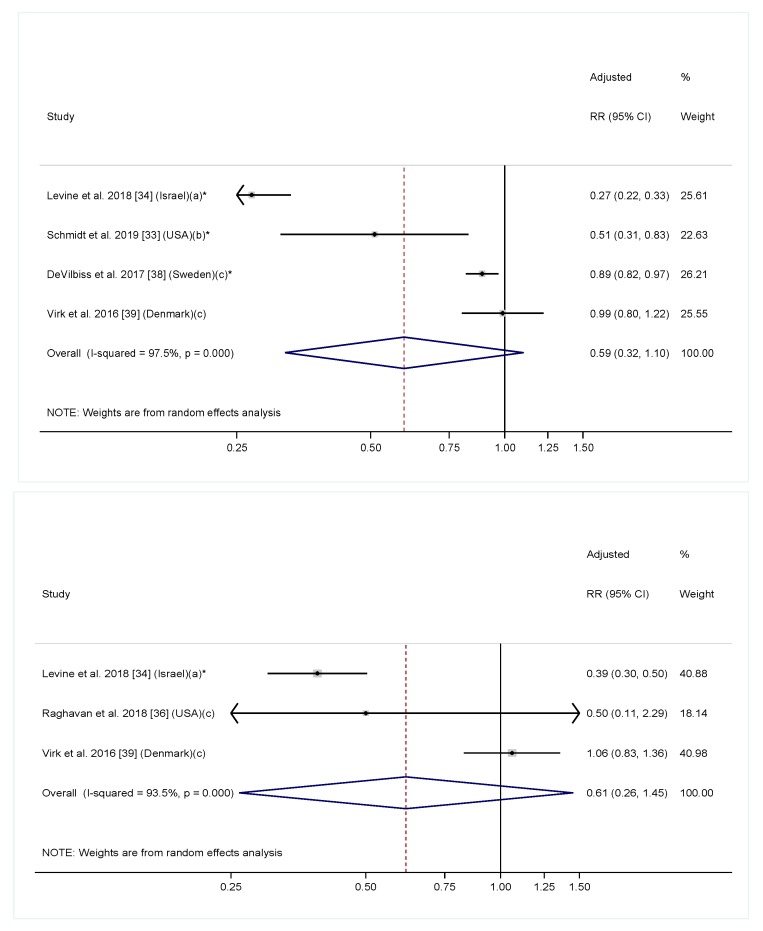
Adjusted relative risk (RRs) of offspring risk of ASD associated with maternal intake of supplement containing folic acid, multivitamin or prenatal vitamin during (top) and before (bottom) pregnancy. The overall effect size was estimated using random effects models weighted by inverse variance of each study. Notes: (a) Intake of supplement containing folic acid vs. no; (b) Folic acid intake ≥600 mcg/day vs. <600 mcg/day; (c) Intake of multivitamin vs. never/rarely. * *p* < 0.05.

In the meta-analysis including six cohort studies reporting a linear relationship between maternal folic acid/multivitamin intake with offspring ASD diagnosis, maternal folic acid/multivitamin intake was significantly associated with a 36% reduction in offspring ASD risk (Figure 3. RR = 0.64, 95% CI: 0.46, 0.90). The heterogeneity across studies was highly significant (*I*^2^ = 96.1%, *p* < 0.001). To further explore the effect of timing on the association, we conducted additional meta-analyses including four studies examining folic acid/multivitamin supplementation during pregnancy only [33,34,38,39], and three studies examining supplementation before pregnancy only [34,36,39]. The overall effect estimates had a similar direction and magnitude compared to the main analysis. However, the confidence intervals were wider, and the associations were not significant for either analyses, likely due to fewer included studies (Figure 4). Of note, most studies that investigated folic acid/multivitamin supplementation during pregnancy were specific to early pregnancy [33,38,39]. Furthermore, studies that investigated supplementation at multiple times during pregnancy consistently found that supplementation in the first or second months of pregnancy, but not later in pregnancy, was associated with lower ASD risk [33,42,44]. We also separately performed meta-analyses for multivitamin or prenatal vitamins and folic acid-specific supplements. The overall effect estimates in both groups were similar to the main analysis, but not significant (Figure S1).

Although folic acid is the most widely hypothesized ingredient in multivitamin related to the risk of offspring ASD risk in existing studies, it cannot be explicitly concluded that folic acid was responsible for the observed association yet. Several biological mechanisms support the potential effect of folic acid on reducing offspring ASD risk though. Changes in maternal folate intake result in altered DNA methylation of some genes such as *IGF2* in offspring [8,9], which has been implicated in neurodevelopmental disorders [56,57]. Furthermore, inadequate folate intake affects offspring brain development in animal models through decreasing progenitor cell proliferation [10] and increasing apoptosis [10,11], leading to impaired short-term memory [11]. On the other hand, more than 50 prenatal multivitamins that include folic acid also contain PUFAs [58], for example, and PUFAs are also known to affect prenatal neurodevelopment (see a summary in the introduction section).

In summary, data from the systematic review suggested an overall inverse association between prenatal folic acid/multivitamin supplementation and ASD risk. We also observed large heterogeneity in the association between folic acid/multivitamin intake and ASD risk across studies, which may reflect differences in background nutrient status [59], supplementation dose [39], and genetic polymorphism in nutrient metabolism [60]. Compared with the two earlier reviews [26,27], the present review included six additional recently published articles, thus, providing more conclusive findings. Future studies should simultaneously examine folic acid and other nutrients contained in multivitamins in order to identify the putative nutrients associated with offspring ASD risk; they should also utilize dietary folate intakes in conjunction with folate biomarkers which are more objective.

#### 3.1.2. Maternal Iron Intake and Offspring ASD Risk

Three studies reported maternal iron supplementation and offspring ASD risk—a in large cohort of 273,107 children in Sweden [38], a small cohort of 332 children who had siblings with ASD in the US [33], and 520 ASD cases and 346 typically developing controls in the US [47]. These studies did not find an association between early pregnancy iron supplementation and ASD, regardless of the specific exposure (iron-specific supplementation, any iron supplementation, total iron supplementation). Only the retrospective study in the US examined iron supplementation later in pregnancy, and it found suggestive evidence of an association between total iron supplementation and iron-specific supplementation in the second and third trimester and a lower ASD risk, which did not reach significance. In addition, it also found a significant association between the exposures during breastfeeding and a lower ASD risk. In summary, studies on iron supplementation intakes in pregnancy and offspring ASD risk are limited. Available data do not suggest an association between prenatal iron supplementation and ASD risk.

#### 3.1.3. Maternal PUFA Intake or Status, Seafood Intake and Offspring ASD Risk

Two studies examined maternal PUFA intake or status in relation to offspring ASD risk or traits. Among 18,045 children in the Nurses’ Health Study II (NHSII) in the US, total and n-6 PUFA intake before pregnancy were all significantly and inversely associated with children’s risk of ASD diagnosis as reported by mothers, whereas n-3 PUFA was not significantly associated with ASD risk [48]. In contrast, among 4624 children in the generation R study in The Netherlands, plasma n-6 PUFA levels in mid-pregnancy were significantly and positively associated with children’s ASD traits measured by Social Response Scale (SRS), the ratio of n-3 to n-6 PUFA was inversely associated with the outcome. Similar to NHSII study, plasma n-3 PUFA levels were not associated with the outcome [49]. Both studies controlled for a comprehensive set of potential confounders including other aspects of diet [48] and dietary supplements [48,49]. Both n-3 and n-6 PUFA are necessary for prenatal brain development [13]; however, an elevated n-6 to n-3 PUFA ratio—characteristic of modern western diet [61]—could potentiate inflammatory processes [62]. Discrepant findings from the two studies may be due to the use of absolute versus relative concentrations of n-3 and n-6 PUFA levels, or differences in the timing of the exposure (before pregnancy vs. mid-pregnancy) and outcome measures (clinical diagnosis vs. trait).

Fish is the main dietary source of DHA—the n-3 PUFA most relevant for brain development [15]. In secondary analysis of the above-mentioned studies on PUFA intake/status, maternal fish intake was not associated with ASD diagnosis/traits [48,49]. In contrast, two other studies suggested a potential inverse association between maternal fish intake and offspring ASD risk [42,51]. Among 1589 children in the Spanish INMA study [51], maternal total seafood intake in the first trimester was associated with lower autistic traits measured by Childhood Asperger Syndrome Test; similar associations were observed for large fatty fish and lean fish. Another study of 108 ASD cases and 108 typically developing controls in China [50] found maternal “habit” (>3 times/week) of eating grass carp reported four to 17 years after delivery to be associated with a lower risk of ASD. However, the validity of the food frequency questionnaire in recalling diet after many years was not reported. Lastly, in one study among 85,176 children in the Norwegian Mother and Child Cohort Study (MoBa) [42], fish oil supplementation before and in early pregnancy was not associated with ASD risk.

In conclusion, findings of the associations between maternal PUFA or fish intake and ASD risk are inconclusive. Heterogeneity in the PUFA measurement or assessment (i.e., absolute vs. relative concentrations, intake vs. status) and ASD (i.e., clinical diagnosis vs. traits, intake vs. status) made it difficult to compare findings across studies. Furthermore, recall bias and potential residual confounding are also of concern.

#### 3.1.4. Maternal Dietary Patterns and Offspring risk of ASD

Two studies reported associations between maternal dietary patterns and ASD risk. Among 325 children in the Newborn Epigenetics Study in the US [52], higher periconception Adherence to the Mediterranean diet was associated with a lower risk of ASD assessed by the Infant Toddler Social Emotional Assessment; the association became non-significant after adjusting for multiple comparisons. Among 374 ASD cases and 354 typically developing controls in the Autism Clinical and Environmental Database in China, maternal recall three to six years after delivery of a “mostly meat” and a “mostly vegetable” dietary pattern during pregnancy were both associated with a higher risk of ASD in children compared to a “both meat and vegetable” dietary pattern [35]. However, it appears that the dietary pattern was only assessed using a single question, and a specific definition of each dietary pattern was not given. Thus, it remains inconclusive if maternal dietary patterns are associated with offspring risk of ASD. More studies on this topic are needed in the future.

### 3.2. Maternal Nutrition and ADHD Risk

Characteristics and main findings of studies assessing the association between maternal nutrition and ADHD are presented in Table 2. Of the 17 studies, 16 are prospective observational study, and one was a randomized controlled trial. These studies were conducted in the UK, France, Spain, The Netherlands, Norway, Denmark, New Zealand, Japan, Brazil, and Mexico.

#### 3.2.1. Maternal Folate Intake/Status, Multivitamin Intake and Offspring ADHD Risk

Three studies examined maternal folic acid supplementation in relation to offspring ADHD risk or symptoms [63,64,65,66,67], with most reporting null findings. Specifically, maternal folic acid-specific supplementation before and in early pregnancy was not associated with clinical diagnosis of hyperkinetic disorders or ADHD medication use in a large study of 35,059 children in the DNBC [65], although it was associated with a lower risk of hyperactivity-inattention problems as measured by the Strength and Difficulties Questionnaire (SDQ) among a subgroup of children followed to age seven. Similarly, maternal intake of any folic acid supplementation before or in early pregnancy was not associated with the risk of the hyperactivity-inattention problem among 6247 children in the Growing Up in New Zealand Study [63] and 420 children in the Menorca cohort in Spain [66].

One study examined maternal folate intake from food in relation to offspring ADHD risk. In the Japanese study including 1199 children in the Kyushu Okinawa Maternal and Child Health Study (KOMCHS) [64], higher intake of folate from food during pregnancy was associated with a non-significant reduction in children’s risk of the hyperactivity-inattention problems (*p*-trend = 0.10), but folic acid supplementation was not accounted for. Lastly, one study examined folate status and total folate intake in relation to ADHD risk. In this small study including 136 children in the UK [67], early pregnancy red blood cell folate concentration and total folate intake from food and supplements, but not late pregnancy intake, were inversely associated with hyperactivity-inattention score. However, unlike the larger studies above, this study only adjusted for a limited set of covariates; residual confounding from maternal lifestyle factors was possible. In summary, no convincing evidence supports an association between folate intake from food or supplements and ADHD risk.

Among the studies previously mentioned, three also assessed the association between maternal multivitamin intake and child ADHD risk [63,65,66]. Overall, the findings are inconsistent. Maternal multivitamin intake was associated with a lower risk of hyperkinetic disorders and ADHD medication in the DNBC [65], but it was not associated with the hyperactivity-inattention problems in the Growing up in New Zealand Study [63]. Maternal intake of multivitamins containing no folic acid was associated with a non-significant reduction in risk of the hyperactivity-inattention problem in the Spanish Menorca cohort (OR = 0.24, 95% CI: 0.05, 1.31) [66]. However, the composition of such multivitamins was not clear. More studies are needed to assess this association further and to examine nutrients other than folate that may be related to ADHD risk.

#### 3.2.2. Maternal PUFA and Seafood Intake and Offspring ADHD Risk

Two studies examined maternal PUFA intake in relation to children’s ADHD risk and findings did not support a significant association. More specifically, among the 1,199 children in the KOMCHS [69], neither maternal intake of total or specific n-3 and n-6 PUFA from food were associated with children’s risk of the hyperactivity-inattention problems. In a randomized controlled trial among 797 children in Mexico [70], maternal DHA supplementation of 400 mg/day from mid-pregnancy to delivery was not associated with clinical risk of ADHD measured by the Conner’s Kiddie Continuous Performance Test (K-CPT) core indicating (>70th percentile). Of note, the ability of the K-CPT to accurately identify children at risk for ADHD has been questioned [79].

Two studies examined maternal seafood intake in relation to children’s risk of hyperactivity-inattention problems. Among 219 children in the UK [71], more frequent intake of oily fish during pregnancy was associated with lower risk of the hyperactivity-inattention problem, whereas intake of all types of fish was not. In contrast, among 8946 children in Avon Longitudinal Study of Parents and Children (ALSPAC) study in the UK [72], seafood intake was not associated with ADHD risk. However, results for oily fish intake were not reported. In summary, studies on the association between maternal PUFA or seafood intake and children’s ADHD risk are inconsistent and provide little evidence for an association; differences in exposure assessments inhibits a clear interpretation of the findings. Future studies need to have a comprehensive assessment of maternal fish, seafood, and PUFA intake/status in relation to offspring ADHD risk.

#### 3.2.3. Maternal Caffeine, Coffee, and Tea Intake and Offspring ADHD Risk

Three cohort studies, including 3485 children in Brazil [74], 3439 children in The Netherlands [75], and 1199 children in Japan [73], consistently found no association between maternal caffeine intake during pregnancy and hyperactivity-inattention problem in children. Across the three studies, the cutoff for the highest intake category was 300–384 mg/day, which is equivalent to three to four and a half cups of coffee/day (a cup coffee is defined as 8 oz). In the meta-analysis pooling all three studies, the overall effect estimate comparing the highest to the lowest category of caffeine intake was null (OR = 0.91, 95% CI: 0.66, 1.27; *I*^2^ = 0.0%, *p* for *I*^2^ = 0.84).

On the other hand, two large prospective cohort studies [76,77] found suggestive evidence that extremely high levels of coffee intake in early pregnancy were associated with an increased risk of ADHD. Among 47,491 children in the DNBC [76], ≥8 cups/day of coffee in early pregnancy (OR = 1.47, 95% CI: 1.18, 1.83), but not late pregnancy (OR = 1.21, 95% CI: 0.95, 1.55), was associated with a significantly increased risk of hyperactivity inattention problems. Among 24,068 children in the Aarhus Birth Cohort in Denmark [77], ≥10 cups/day of coffee in early pregnancy was also associated with a substantial but non-significant increase in the risk for hyperkinetic disorders and ADHD diagnosis (OR = 2.3, 95% CI: 0.9, 5.9). Less coffee intake (<8 cups/day in the DNBC and <10 cups/day in the Aarhus Birth Cohort) was not associated with ADHD risk in either study. Of note, only about 3% of women had extremely high intake levels in both studies [76,77], and they appeared to be highly selective group, characterized by lower social class, much higher rate of cigarette smoking, and higher rate of hyperactivity themselves [76]. While the two studies adjusted for selected socioeconomic, lifestyle and psychological characteristic of the women [76,77], residual confounding from unmeasured variables cannot be ruled out. On the other hand, it is possible that only extreme levels of caffeine consumption affect fetal neurodevelopment [20]. The extreme levels of coffee intake were not captured in the studies examining caffeine intake. Taken together, these studies suggest a null association between moderate coffee intake and offspring ADHD risk. Further evaluation of extremely high levels of maternal coffee intake and ADHD risk is needed.

#### 3.2.4. Maternal Dietary Patterns and Offspring ADHD Risk

Two cohort studies [56,78] suggested an association between poor dietary quality during pregnancy and increased risk of ADHD or ADHD symptoms in children. Among 1242 children in the EDEN mother-child cohort in France [56], the lowest quartile of “healthy dietary patterns” (i.e., high intake in fruit, vegetables, fish, and whole grain cereals) and the highest quartile of “western dietary pattern” (i.e., high intake in processed and snacking foods) were both associated with increased risk of hyperactivity-inattention trajectory between age three and eight years. In the ALSPAC [56], an “unhealthy dietary pattern” (i.e., high fat and sugar) score was positively associated with ADHD symptoms among 83 youths with early-onset persistent conduct problems, but not associated with ADHD symptoms among the 81 youths with low conduct problems; the association among youths with early-onset persistent conduct problems was mediated by higher levels of *IGF2* DNA methylation at birth. Of note, *IGF2* gene has important roles in regulating placental and fetal growth; genetic and epigenetic changes in the *IGF2* gene and changes in IGF2 hormonal levels has been linked to altered development in the cerebellum and hippocampus, both of which are relevant to ADHD [56]. The significant association between maternal dietary quality and children’s ADHD risk should be further evaluated.

## 4. Methodological Issues and Future Directions

Two major methodological concerns among studies of maternal nutrition and offspring neurodevelopmental disorders merit further discussion. The first is potential measurement error in the exposure assessment. Studies that comprehensively characterized nutrients from both food and supplements are limited. This is relevant as the effect of the supplementation may depend on baseline nutrient sufficiency status before supplementation, as well as the supplementation dose. Similarly, the effects of a nutrient from food may be overwhelmed by supplementation. Moreover, most studies examined maternal nutritional exposures using questionnaires, including food frequency questionnaires, which are prone to recall bias. To address these concerns, future studies need to prospectively quantify nutrient intakes from both foods and dietary supplements, which may help to establish potential dose-response relations and sufficient dose to prevent offspring neurodevelopmental disorders. Future studies should also consider quantifying nutrient status using biomarkers, which complement dietary information from questionnaires and provide a source of validation.

A second methodological concern with regard to existing studies is potential bias due to residual confounding. Many maternal demographic, lifestyle, and psychosocial factors that contribute to poorer maternal nutritional status may also contribute to the risk of neurodevelopmental disorders in children [4,80]. Smaller studies frequently lack the power to adjust for a comprehensive list of potential confounders. Even in large studies where such adjustments were made, residual confounding from unknown risk factors are still possible, and a causal link cannot be established. Alternative study designs may help to ameliorate this concern and strengthen the evidence. For example, variants in *MTHFR* gene encoding methylenetetrahydrofolate reductase affect vulnerability to low folate intake [55]; variants in *FADS* gene encoding fatty acid desaturase are determinants of long change PUFA status [81]. These genetic variants have been found to modify the association between maternal intake of respective nutrients and offspring neurodevelopmental outcomes [15,44]. Futures studies may use these variants to implement Mendelian randomization in observational studies to further evaluate a potential causal effect of nutrients on offspring outcomes.

Besides these major methodological concerns, several others may merit consideration in designing future studies. First, the effect of a nutrient on neurodevelopmental disorders may vary by fetal developmental period, reflecting the underlying developmental processes affected. Many existing studies did not investigate the timing of the exposure explicitly. Future studies should have explicit hypotheses about the window of sensitivity, and use it inform study design and implementation and the interpretation of results. Longitudinal assessment of the exposures throughout relevant periods would be particularly suitable to identify the most relevant window of sensitivity to the nutrient. Knowledge of the sensitive window of exposure is essential for establishing evidence and designing interventions. Second, epigenetic modification is postulated to be major mechanism by which early life nutrition contributes to risk of health and disease later in life [82], and maternal intake of nutrients such as folate and DHA has been linked to differential DNA methylation patterns [8]. Two studies have reported promising findings where maternal diet has been simultaneously linked to altered methylation of specific genes and neurodevelopmental disorders [52,56]. More studies incorporating epigenetic data are needed to better understand the mechanism.

## 5. Conclusions

Maternal nutrition is a potentially modifiable factor important for fetal neurodevelopment. In the past decade, a body of research emerged examining the impact of maternal nutritional status and offspring developmental disorders. To our knowledge, this is the first comprehensive and systematic review of maternal nutrition during the preconception and perinatal period and offspring risk of neurodevelopmental disorders. This review included studies on maternal intake (or status) at multiple levels: nutrients (i.e., vitamins, minerals, PUFA, multivitamins), food (i.e., fish), and dietary patterns in relation to children’s risk of ASD and ADHD. Findings from the review supported an inverse association between maternal folic acid or multivitamin intake and children’s risk of ASD, although large heterogeneity existed across studies. Data on associations of other dietary factors and ASD, ADHD and related outcomes are inconclusive and warrant future investigation. Future studies that comprehensively quantify maternal nutrient intake from both food and supplements and integrate more objective measures of biomarkers reflecting intake and metabolism are warranted. In addition, incorporating genetic variants related to nutrient metabolism shall enable Mendelian randomization analyses to inform causal inferences and a better understanding of gene-diet interactions in relation to neurodevelopmental disorders. Understanding the sensitive window of exposure and gene-diet interactions may help inform precise intervention and prevention.

## Figures and Tables

**Table 1:** Studies on maternal nutrition and offspring ASD risk or traits.

Source (Country)	Design & Sample	Maternal Exposures	Offspring Outcomes	Findings	Covariates
**Vitamins and Minerals**					
**Folate and Multivitamin-ASD Diagnosis or Cutoff**					
Schmidt et al. 2019 (USA) [33]	Prospective cohort; 332 children who were younger sibling of children with ASD in the MARBLES study	Vitamin and supplement use for the 6 months preconception and each month during the pregnancy assessed in interviews in the first and second halves of pregnancy and after birth	ASD assessed by ADOS at 3 years of age	Prenatal vitamin in the first month of pregnancy was associated with lower ASD risk (RR = 0.50, 95% CI: 0.30 to 0.81) *. Folic acid supplement ≥600 mcg/day in the first month of pregnancy was associated with lower ASD risk (RR = 0.38, 95% CI: 0.16, 0.90) *	Maternal education. The folic acid model further adjusted for iron intake
Levine et al. 2018 (Israel) [34]	Prospective cohort; 45,300 children	Intake of folic acid and multivitamin supplements before and during pregnancy was coded using ATC from the prescription registry	ASD diagnosis identified from health care registers from the Meuhedet health care organization; children were 10 years old	Folic acid and/or multivitamin both before (RR = 0.39, 95% CI: 0.30, 0.50) * and during (RR = 0.27, 95% CI: 0.22, 0.33) * pregnancy were associated with lower risk of ASD. Results on folic acid supplements and multivitamin as two separate exposures were consistent with the main findings	sex, birth year, socioeconomic status, a maternal and paternal psychiatric diagnosis by childbirth, maternal and paternal age at childbirth, and parity
Li et al. 2018 (China) [35]	Retrospective case-control; 374 ASD and 354 TD in the ACED	Food preference and supplement use preconception and during pregnancy assessed 3–6 years after delivery	ASD identified from special education schools; TD identified from ordinary schools; children were 3–6 years of age	Maternal folic acid supplementation before pregnancy was not associated with ASD risk (OR = 0.95, 95% CI: 0.61, 1.50); Maternal folic acid supplementation during pregnancy was associated with lower ASD risk (OR = 0.64, 95% CI: 0.41, 1.00) *	Child’s and parental age, child’s gender, parental education, maternal BMI before conception and delivery, premature delivery, and intake of other supplements
Raghavan et al. 2018 (USA) [36]	Prospective cohort; 1257 children in the Boston Birth Cohort	Multivitamin supplement intake before pregnancy and in each trimester assessed 1–3 days after delivery; plasma folate and vitamin B12 levels measured 1–3 days after delivery	ASD identified from electronic medical records in Boston Medical Center	Before pregnancy, multivitamin supplement was not associated with ASD risk (HR = 0.5, 95% CI: 0.1, 2.1). In the first trimester, ≤2 (HR = 3.4, 95% CI: 1.6, 7.2) * and >5 (HR = 2.3, 95% CI: 1.2, 3.6) * times/week of multivitamin supplement were both associated with higher risk of ASD compared to 3–5 times/day. Findings are similar at second and third trimester. Very high serum folate (HRs [95% CI] for decile 1 and 10 vs. the rest were 1.2 [0.5, 2.8] and 2.5 [1.3, 4.6]) * and vitamin B12 (HRs [95% CI] for decile 1 and 10 vs. the rest were 0.7 [0.3, 1.7] and 2.5 [1.4, 4.5]) * concentrations were both associate with higher ASD risk.	Maternal age, education, parity, BMI, smoking status, diabetes status, race, and *MTHFR* genotype, offspring gestational age, sex, and year of birth
Strom et al. 2018 (Denmark) [37]	Prospective cohort; 92,676 children in the DNBC	Folic acid supplementation and folate intake from food in the previous 4 weeks assessed by FFQ at GW 25	ASD cases identified in Danish Central Psychiatric Research Registry and the Danish National Patients Registry	Folic acid supplement at GW −4 to 8 was not associated with ASD risk (HR = 1.04, 95% CI: 0.94, 1.19). Folic acid supplement in mid pregnancy was not associated with ASD risk (HRs [95% CI] for <400 and ≥400 mcg/day vs. no intake was 1.01 [0.76, 1.34] and 0.98 [0.75, 1.29]). Folate from food was not associated with SD risk (HRs [95% CI] for quintile 2–5 were 0.82 [0.67, 1.01], 0.96 [0.78, 1.17], 0.85 [0.69, 1.04] and 0.94 [0.77, 1.16])	Maternal age, paternal age, parity, maternal smoking during pregnancy, maternal education, family socioeconomic status, whether the pregnancy was planned, maternal prepregnancy BMI and sex of the child
DeVilbiss et al. 2017 (Sweden) [38]	Prospective cohort; 273,107 children in the Stockholm youth cohort	Supplement use at the first antenatal visit coded in ATC from medical birth registry	ASD identified from computerized registers covering all pathways of ASD diagnosis and care in Stockholm County; children were 4–15 years old	Multivitamin supplement was associated with lower risk of ASD compared to no vitamin/mineral supplement (OR = 0.89, 95% CI: 0.82, 0.97) *. Folic acid supplement alone (OR = 1.27, 95% CI: 1.01, 1.60), iron supplement alone (0.96, 95% CI: 0.90, 1.03) and combined folic acid and iron supplement (OR = 0.92, 0.83 to 1.02) were not associated with ASD risk	Child characteristics (sex, birth year, and years resided in Stockholm County), socioeconomic indicators (education, family income, and maternal birth country), maternal characteristics (age, BMI, parity, smoking status), medication use during pregnancy (antidepressants or antiepileptics), and maternal neuropsychiatric conditions (anxiety disorders, autism, bipolar disorder, depression, epilepsy, intellectual disability, non-affective psychotic disorders, and stress disorders)
Virk et al. 2016 (Denmark) [39]	Prospective cohort; 35,059 children in the DNBC	Folic acid and multivitamin supplementations from 4 weeks preconception to GW 8 assessed by questionnaire at GW 12	ASD identified from National Hospital Register; children were 10 years of age	Folic acid supplement at GW −4 to 8 was not associated with ASD risk (RR = 1.06, 95% CI: 0.82, 1.36). Multivitamin supplement was not associated with ASD risk (RR = 1.00, 95% CI: 0.82, 1.22)	Maternal age; maternal smoking and alcohol consumption during pregnancy; household socioeconomic status-examined more but none changed estimates more than 5%
Braun et al. 2014 (USA) [40]	Prospective cohort; 209 children in the HOMA study	Current vitamin supplementation assessed by interviews at GW 14–39; whole blood folate concentrations measured at GW 11–21	Autistic-behaviors assessed by SRS at 4–5 years of age; scores >60 were defined as abnormal	Vitamin supplementation was associated with lower risk of failed SRS test (weekly/daily vs. never/rare intake: OR = 0.26, 95% CI: 0.08, 0.89) *. Whole blood folate concentrations were not associated with ASD risk (OR per SD = 1.42, 95% CI: 0.81, 2.49)	Whole blood folate concentration, maternal age, race, education, household income, marital status, employment during pregnancy, insurance status, depressive symptoms, serum cotinine concentrations, food security, and fresh fruit/vegetable intake
Nilsen et al. 2013 (Norway) [41]	Prospective cohort; 89,836 children in the MoBa	Folic acid intake before and/or during pregnancy recorded in Medical Birth Registry of Norway	ASD identified in Norwegian Patient Registry; all children were 3 years of older	Folic acid supplement was associated with lower ASD risk (OR = 0.86, 95% CI: 0.78, 0.95) *	Year of birth, maternal age, paternal age, marital status, and parity, hospital size
Suren et al. 2013 (Norway) [42]	Prospective cohort; 85,176 children in the MoBa	Folic acid and other supplementations from 4 weeks preconception to GW 8 assessed by questionnaire at GW 12	ASD identified through questionnaire screening at 3, 5 and 7 years, professional and parental referral, and the Norwegian Patient Registry when children were 3–10 years of age	Folic acid supplement was associated with lower ASD risk (OR = 0.61, 95% CI: 0.41, 0.90) *. Supplements with folic acid were associated with lower ASD risk, supplements without folic acid were not	Adjusted for year of birth, maternal education level, and parity
DeSoto et al. 2012 (USA) [43]	Retrospective case-control; 256 ASD and 752 TD in the Vaccine Safety Datalink project	Folic acid intake from prenatal vitamins	ASD identified based on medical record	Folic acid supplement/prenatal multivitamin was associated with higher ASD risk (OR = 2.34, 95% CI: 1.14, 4.82) *	Child and family characteristics (e.g., maternal age, birth weight, poverty ratio, birth order, breast feeding duration), maternal prenatal health care/seeking behavior (e.g., adequacy of prenatal care, cholesterol screen, pap smear, prenatal alcohol use, prenatal viral infections), and child medical conditions (e.g., anemia, pica)
Schmidt et al. 2012 (USA) [44]	Retrospective case-control; 429 ASD and 278 TD in CHARGE study	Folic acid intake from supplements and fortified cereals in each month from 3 months before conception to delivery assessed by interviews at 2–5 years after delivery	ASD identified from the California Regional Center System; matched TD identified from state birth files; children were 2 to 5 years old	Higher folic acid intake in the first trimester was associated with lower ASD risk (ORs [95% CI] for <500, 500–799, 800–1000 and >1000 mcg/day vs. no intake were 0.35 [0.10 1.24], 0.27 [0.06 1.36], 0.25 [0.05 1.22] and 0.18 [0.04 0.94]) *	Adjusted for maternal educational level, child’s birth year, and log-transformed total iron and vitamin E from supplements and cereals; estimates were not substantially different when further adjusted for log-transformed vitamin B-12, vitamin B-6, vitamin C, vitamin D, or calcium from supplements and cereals or when adjusted for prenatal vitamin use in the first month of pregnancy
Schmidt et al. 2011 (USA) [45]	Retrospective case-control; 288 Autism and 278 TD in CHARGE study	Vitamin use in each month from 3 months before conception to delivery assessed by interviews at 2–5 years after delivery	Autism identified from the California Regional Center System; matched TD identified from state birth files; children were 2 to 5 years old	Prenatal vitamin supplement was associated with lower autism risk (OR [95% CI] for any use was 0.61 [0.39, 0.97]; ORs [95% CI] for irregular or <4 days/week, 4 days/week-daily, and > daily vs. no use were 0.96 [0.18–5.0], 0.61 [0.38–0.98] and 0.51 [0.19–1.4], p-trend = 0.002) * Multivitamin supplement was not associated with ASD risk [OR 1.2, 95% CI [0.51, 2.60]).	Maternal education and the child’s year of birth
Folate and multivitamin-ASD traits					
Steenweg-de Graaff et al. 2015 (The Netherlands) [46]	Prospective cohort; 3893 children in the Generation R study	Plasma folate concentrations at GW 10–17; folic acid supplementation from preconception to early pregnancy assessed by questionnaire early in pregnancy	Autistic traits using the SRS short form at 6 years of age; scores >95th percentile defined as abnormality	Folic acid supplement starting before pregnancy (beta per SD = −0.042, 95% CI: −0.068, −0.017) *, before GW 10 (beta per SD = −0.041, 95% CI: −0.066, -0.016) *, and after GW 10 (beta per SD = −0.057, 95% CI: −0.089, −0.025) * in pregnancy were all associated with fewer autistic traits. Higher serum folate concentration was not associated with fewer autistic traits (beta per SD = −0.007, 95% CI: −0.016, 0.001), nor the risk of being a probable ASD case (OR per SD = 1.03, 95% CI: 0.76, 1.39).	Gestational age at venipuncture, gender and age of the child, maternal psychopathology, education and family income
Iron-ASD diagnosis or cutoff					
Schmidt et al. 2019 (USA) [33]	Prospective cohort; 332 children who were younger sibling of children with ASD in the MARBLES	Vitamin and supplement use for the 6 months preconception and each month during the pregnancy assessed in interviews in the first and second halves of pregnancy and after birth	ASD assessed by ADOS at 3 years of age	Iron supplement in the first month of pregnancy was not associated with ASD risk (RR = 1.47, 95% CI: 0.61, 3.55)	Maternal education and folic acid supplement
DeVilbiss et al. 2017 (Sweden) [38]	Prospective cohort; 273,107 children in the Stockholm Youth Cohort	Supplement use at the first antenatal visit coded in ATC from medical birth registry	ASD identified from computerized registers covering all pathways of ASD diagnosis and care in Stockholm County; children were 4–15 years old	Folic acid supplement alone (OR = 1.27, 95% CI: 1.01, 1.60), iron supplement alone (OR = 0.96, 95% CI: 0.90 to 1.03) and combined folic acid and iron supplement (OR = 0.92, 95% CI: 0.83, 1.02) were not associated with ASD risk	Child characteristics (sex, birth year, and years resided in Stockholm County), socioeconomic indicators (education, family income, and maternal birth country), maternal characteristics (age, body mass index, parity, smoking status), medication use during pregnancy (antidepressants or antiepileptics), and maternal neuropsychiatric conditions (anxiety disorders, autism, bipolar disorder, depression, epilepsy, intellectual disability, non-affective psychotic disorders, and stress disorders)
Schmidt et al. 2014 (USA) [47]	Retrospective case-control; 520 ASD and 346 TD in CHARGE study	Iron supplementation in each month from 3 months before conception to delivery assessed by interviews at 2–5 years after delivery	ASD identified from the California Regional Center System; matched TD identified from state birth files; children were 2 to 5 years old	Iron-specific supplement before and during pregnancy was not associated with ASD risk (ORs [95% CI] for gestational months −3 to 9 were 0.89 [0.19, 4.13], 0.89 [0.19, 4.13], and 0.78 [0.21, 2.84], 0.67 [0.29, 1.55], 0.72 [0.37, 1.39], 0.71 [0.40, 1.27], 0.64 [0.39, 1.04], 0.65 [0.41, 1.04], 0.66 [0.43, 1.02], 0.73 [0.48, 1.13], 0.77 [0.49, 1.20] and 0.64 [0.39, 1.03]). Total iron supplement before and during pregnancy were not associated with ASD risk	Maternal folic acid intake, home ownership, child’s birth year. The model on total iron supplement was adjusted for folic acid
**Calcium-ASD diagnosis or cutoff**					
Li et al. 2018 (China) [35]	Retrospective case-control; 374 ASD and 354 TD in the ACED	food preference and supplement use preconception and during pregnancy assessed 3–6 years after delivery	ASD identified from special education schools; TD identified from ordinary schools; children were 3–6 years of age	Maternal calcium supplementation before pregnancy was associated with lower ASD risk (OR = 0.48, 95% CI: 0.28, 0.84) *; during pregnancy, it was not associated with ASD risk (OR = 1.11, 95% CI: 0.70, 1.75)	Child’s and parental age, child’s gender, parental education, maternal BMI before conception and delivery, premature delivery, and intake of other supplement
PUFA and seafood					
PUFA-ASD diagnosis or cutoff					
Lyall et al. 2013 (USA) [48]	Prospective cohort; 18,045 children in NHSII	Fat intake before pregnancy reported in the year assessed by FFQ after delivery	Autism, Asperger Syndrome, or PDD diagnosis reported by mothers at 4 years of age	Higher total PUFA from food (RRs [95% CI] for quartile 2–4 were 0.74 [0.55, 1.00], 0.73 [0.54, 0.99] and 0.77 [0.47, 1.26], *p*-trend = 0.05) * was associated with lower ASD risk. Total n-3 PUFA (0.98 [0.73, 1.32], 0.78 [0.57, 1.06] and 0.90 [0.66, 1.22], *p*-trend = 0.12), ALA (0.91 [0.67, 1.23], 0.86 [0.64, 1.16] and 0.80 [0.58, 1.08], *p*-trend = 0.14), EPA (1.11 [0.80, 1.54], 1.13 [0.79, 1.61] and 1.07 [0.76, 1.51], p-trend = 0.97), and DHA (1.05 [0.78, 1.42], 0.95 [0.69, 1.31] and 1.07 [0.79, 1.45], *p*-trend = 0.74) from food were not associated with ASD risk. Total n-6 PUFA (1.01 [0.75, 1.36], 1.01 [0.75, 1.36] and 0.66 [0.47, 0.92], *p*-trend = 0.01) * and LA (1.01 [0.76, 1.35], 0.86 [0.64, 1.16], 0.86 [0.64, 1.16] and 0.66 [0.48, 0.92], *p*-trend = 0.008) * from food were associated with lower ASD risk; AA (0.98 [0.73, 1.32], 0.78 [0.57, 1.06] and 0.79 [0.58, 1.09], *p*-trend = 0.09) were not associated with ASD risk	Adjusted for total energy intake, maternal age, child’s year of birth, income level, race, body mass index, and prepregnancy smoking status. Removal of adjustment for smoking did not affect results. Additional adjustment for intake of protein, whole grains, alcohol, fruit, and vegetables, as well as for multivitamin use, physical activity, child birth order, and maternal pregnancy complications did not materially alter the results
PUFA-ASD traits					
Steenweg-De Graaff et al. 2016 (The Netherlands) [49]	Prospective cohort; 4624 children in the Generation R study	Plasma fatty acid profiles measured before GW 25	Autistic traits assessed by SRS at 6 years of age	N-3 PUFA percentage was not associated with autistic trait (beta per SD = −0.002, 95% CI: −0.011, 0.006). Higher n-6 PUFA percentage was associated with fewer autistic traits (beta per SD = 0.011, 95% CI: 0.002, 0.020) *. Higher n-3 to n-6 ratio was associated with fewer autistic traits (beta per SD = −0.009, 95% CI: −0.017, −0.001) *	Gestational age at venipuncture, sex, and age of the child at assessment, maternal IQ, prepregnancy body mass index, educational level, national origin, age at enrollment, psychopathology score in mid-pregnancy, smoking, alcohol consumption, and folic acid supplement use during pregnancy, family income, child day-care attendance, and paternal educational level, national origin, and psychopathology score
Seafood-ASD diagnosis or cutoff					
Gao et al. 2016 (China) [50]	Retrospective case-control; 108 ASD and 108 TD	Fish intake 6 months before pregnancy until delivery, assessed by FFQ 4–17 years after delivery	ASD identified from the registry of special education schools; matched TD identified from ordinary schools; children were 4–17 years old	Maternal no habit of eating grass carp was associated with higher risk of ASD (OR = 3.59, 95% CI: 1.22, 10.51) *	Maternal habit of eating grass carp, parental habit of eating hairtail, income level at childbirth, current income level. Paternal education, maternal education; matched on child age and sex
Lyall et al. 2013 (USA) [48]	Prospective cohort; 18,045 children in NHSII	Fish intake before pregnancy reported in the year assessed by FFQ after delivery	Autism, Asperger Syndrome, or PDD diagnosis reported by mothers at 4 years of age	Fish intake was not associated with ASD risk (RR [95% CI] for <1, 1 and >1 time/week were 1.10 [0.73, 1.66], 0.99 [0.65, 1.50] and 1.02 [0.59, 1.75])	Adjusted for total energy intake, maternal age, child’s year of birth, income level, race, body mass index, and prepregnancy smoking status. Removal of adjustment for smoking did not affect results. Additional adjustment for intake of protein, whole grains, alcohol, fruit, and vegetables, as well as for multivitamin use, physical activity, child birth order, and maternal pregnancy complications did not materially alter the results
Seafood-ASD traits					
Julvez et al. 2016 (Spain) [51]	Prospective cohort; 1589 children in the INMA study	Seafood intake in the first trimester assessed by interviews with FFQ at GW 10–13	Autism spectrum traits assessed by the Childhood Asperger Syndrome Test based on parent report at 5 years of age	Higher total seafood intake was associated with fewer autistic traits (beta [95% CI] for quintile 2–5 vs. 1 were −0.42 [−0.90, 0.07], −0.45 [−0.95, 0.05], -0.61 [−1.12, −0.11], and −0.55 [−1.06, −0.04], *p*-trend = 0.04) *. Large fatty fish and lean fish were both associated with fewer autistic traits, whereas small fatty fish and shellfish were not associated	Sex of the child, age during testing, cohort, quality of the test, and maternal energy intake during pregnancy, child’s birth weight, gestational age, duration of breastfeeding, maternal age, educational level, social class, prepregnancy body mass index, parity, and country of origin/birth
Steenweg-De Graaff et al. 2016 (The Netherlands) [49]	Prospective cohort; 4624 children in the Generation R study	Fish intake in the past 3 months assessed by FFQ in early pregnancy	Autistic traits assessed by SRS at 6 years of age	Fish intake was not associated with autistic trait (beta = −0.022, 95% CI: −0.055, 0.010)	Gestational age at venipuncture, sex, and age of the child at assessment, maternal IQ, prepregnancy body mass index, educational level, national origin, age at enrollment, psychopathology score in mid-pregnancy, smoking, alcohol consumption, and folic acid supplement use during pregnancy, family income, child day-care attendance, and paternal educational level, national origin, and psychopathology score
Fish oil-ASD diagnosis or cutoff					
Suren et al. 2013 (Norway) [42]	Prospective cohort; 85,176 children in the MoBa	Folic acid and other supplementations from GW −4 to 8 weeks assessed by questionnaire at GW 12	ASD identified through questionnaire screening at 3, 5 and 7 years, professional and parental referral, and the Norwegian Patient Registry when children were 3–10 years of age	Fish oil supplement at GW −4 to 8 was not associated with ASD risk (OR = 1.29, 95% CI: 0.88, 1.89)	Adjusted for year of birth, maternal education level, and parity
Fruit					
Fruit-ASD diagnosis or cutoff					
Gao et al. 2016 (China) [50]	Retrospective case-control; 108 ASD and 108 TD	Fruit intake 6 months before pregnancy until delivery, assessed by FFQ 4–17 years after delivery	ASD identified from the registry of special education schools; matched TD identified from ordinary schools; children were 4–17 years old	Maternal no habit of eating fruits was associated with higher risk of ASD (OR = 2.42, 95% CI: 1.24, 4.73) *	maternal habit of eating grass carp, parental habit of eating hairtail, income level at childbirth, current income level. Paternal education, maternal education; frequency matched on child age and sex
Dietary patterns					
Dietary patterns-ASD diagnosis or cutoff					
House et al. 2018 (USA) [52]	Prospective cohort; 325 children in the NEST study	adherence to Mediterranean diet periconception assessed by FFQ in the first trimester of pregnancy or at enrollment	ASD index from ITSEA administered by a parent, caregiver or staff at 1–2 years of age	Adherence to Mediterranean diet was associated with lower ASD risk (ORs [95% CI] for tertile 2 and 3 vs. 1 were 0.46 [0.23, 0.90] and 0.35 [0.15, 0.80]) *. However, the trend was not significant after FDA adjustment (*p* = 0.09)	Breastfeeding at least 3 months, age of child at behavioral assessment, maternal fiber intake, total calories, folate, education, diabetes, obesity, smoking, and age, as well as paternal age and child parity, premature birth, weight, race, and child sex
Li et al. 2018 (China) [35]	Retrospective case-control; 374 ASD and 354 TD in the ACED	“Mostly meat”, “mostly vegetable” or “both meat and vegetable” dietary patterns assessed in questionnaires 3–6 years after delivery	ASD identified from special education schools; TD identified from ordinary schools; children were 3–6 years of age	Before pregnancy, maternal mostly meat (OR = 4.01, 95% CI:1.08, 14.89) * and mostly vegetable dietary pattern (OR = 2.23, 95% CI: 1.01, 4.95) * were both associated with higher ASD risk compared to both meat and vegetable dietary pattern. During pregnancy, they were not associated with ASD risk (ORs [95% CI] were 1.36 [0.29, 6.32] and 1.20 [0.53, 2.68])	Child’s and parental age, child’s gender, parental education, maternal BMI before conception and delivery, premature delivery, and other maternal dietary patterns

* Statistically significant findings (*p*-Value < 0.05). Abbreviates of research studies: ACED—Autism Clinical and Environmental Database; CHARGE—Childhood Autism Risks from Genetics and the Environment; DNBC—Danish National Birth Cohort; HOME—Health Outcomes and Measures of the Environment; INMA—INfancia y Medio Ambiente; ITSEA—Infant Toddler Social Emotional Assessment; MARBLES—Markers of Autism Risk in Babies—Learning Early Signs; NHSII—Nurses’ Health Study II; MoBa—Norwegian Mother and Child Cohort Study. Other abbreviates: ADOS—the Autism Diagnostic Observation Schedule; ASD—autism spectrum disorder; ATC—Anatomical Therapeutic Chemical; BMI—body mass index; CI—confidence interval; FFQ—food frequency questionnaire; GW—gestational week; HR—hazard ratio; MTHFR—methylenetetrahydrofolate reductase; OR—odds ratio; PDD—pervasive developmental disorders; RR—relative risk; SD—standard deviation; SRS—social response scale; TD—typically developing.

**Table 2:** Studies on maternal nutrition and offspring ADHD risk or symptoms.

Source (Country)	Design & Sample	Maternal Exposures	Offspring Outcomes	Findings	Covariates
**Vitamins and Minerals**					
**Folate and Multivitamin-ADHD Diagnosis or Cutoff**					
D’Souza et al. 2019 (New Zealand) [63]	Prospective cohort; 6246 children in the Growing Up in New Zealand Study	Folic acid and multivitamin supplementation before pregnancy, during the first trimester, and after the first trimester assessed in interviews in late pregnancy	Hyperactivity-inattention symptoms assessed by SDQ using mothers’ report at 2 years of age; clinical cutoff was used to define abnormality	Folic acid intake was not associated with ADHD risk (ORs [95% CI] for first trimester only and no intake vs. intake both before pregnancy and at first trimester were 0.98 [0.74, 1.31] and 0.88 [0.57, 1.34]). Multivitamin was not associated with ADHD risk (OR = 0.97, 95% CI: 0.75, 1.24)	Mother’s ethnicity, mother’s education, mother’s age when pregnant, child’s gestational age, child’s birth weight, child’s gender, parity, planned pregnancy, mother in paid employment, area-level deprivation, and rurality
Miyake et al. 2018 (Japan) [64]	Prospective cohort; 1199 children in the KOMCHS	Folate and other B-vitamin intake from food in the past month assessed by FFQ at GW 5 to 39	Hyperactivity-inattention symptoms assessed by SDQ using mothers’ report at 5 years of age; clinical cutoff was used to define abnormality	Folate from food was not associated with hyperactivity-inattention problem (ORs [95% CI] for quartile 2–4 were 0.75 [0.46, 1.21], 0.66 [0.40, 1.07], and 0.69 [0.42, 1.12], *p*-trend = 0.10). Vitamin B12 (ORs [95% CI] for quartile 2–4 were 0.80 [0.49, 1.29], 0.99 [0.61, 1.61] and 0.81 [0.50, 1.32], *p*-trend = 0.60) and B2 (ORs for quartile 2–4 were 1.09 [0.68, 1.75], 1.03 [0.64, 1.66] and 0.61 [0.36, 1.03], *p*-trend = 0.08) from food were not associated hyperactivity-inattention problem. Higher vitamin B6 from food was associated with lower risk of hyperactivity-inattention problem (ORs for quartile 2–4 were 0.76 [0.48, 1.21], 0.58 [0.36, 0.94] and 0.57 [0.34, 0.94], *p*-trend = 0.01) *	Maternal age, gestation at baseline, region of residence at baseline, number of children at baseline, maternal and paternal education, household income, maternal depressive symptoms during pregnancy, maternal alcohol intake during pregnancy, maternal vitamin B complex supplement use during pregnancy, maternal smoking during pregnancy, child’s birth weight, child’s sex, breastfeeding duration, and smoking in the household during the first year of life.
Virk et al. 2018 (Denmark) [65]	Prospective cohort; 35,059 children in the DNBC	Folic acid and multivitamin supplementations from GW −4 to 8 assessed by questionnaire at GW 12	Hyperkinetic disorder and treatment for ADHD were identified from National Patient Register; children were 7 years of age. Hyperactivity-inattention symptoms assessed by SDQ at age 7 years based on parent reports, and a score ≥7 was defined as abnormal	Folic acid supplement was not associated with risk of hyperkinetic disorder diagnosis (HR = 0.87, 95% CI: 0.54, 1.41) or ADHD medication (HR = 0.96, 95% CI: 0.68, 1.37). Maternal multivitamin use was associated with lower risk of hyperkinetic disorder diagnosis (HR = 0.70, 95% CI: 0.52, 0.96) *, ADHD medication (HR = 0.78, 95% CI: 0.62, 0.98) *	Maternal age, household socio-economic status, maternal smoking and alcohol consumption during pregnancy, maternal prepregnancy body mass index, birth year, and offspring sex
Julvez et al. 2009 (Spain) [66]	Prospective cohort; 420 children in the Menorca cohort	Current folic acid and vitamin supplementations assessed by interviews at GW 12	ADHD assessed by ADHD Rating Scale-IV based on teacher report at 4 years of age; scores >80th percentile was defined abnormal	Folic acid with or without other vitamins compared to no folic acid or vitamins was not associated with ADHD risk (OR = 0.74, 95% CI: 0.38, 1.47). Vitamins without folic acid compared to no folic acid or vitamins was not associated with ADHD risk (OR = 0.26, 95% CI: 0.05, 1.31)	Parental social class and level of education, mother’s parity at child’s age four, mother’s marital status, maternal tobacco smoking during pregnancy, maternal intake of supplementary calcium and iron at the same time as study determinants, gestational age at interview, child’s gender, child’s duration of breast feeding, child’s age and school season during test assessment, evaluator and child’s home location at age four
Folate and multivitamin-ADHD symptoms					
Schlotz et al. 2010 (UK) [67]	Prospective cohort; 139 children	Total folate intake from foods and supplements during early pregnancy assessed by FFQ at GW 14, and during late pregnancy assessed at GW 28	Hyperactivity-inattention symptoms assessed by SDQ based on mothers’ report at 8 years	Maternal red cell folate concentration (beta per SD = −1.23, 95% CI: −2.20, −0.26) * and total folate intake from food and supplements (beta per SD = −0.75, 95% CI: −1.39, −0.11) * in early pregnancy were both associated with fewer hyperactivity-inattention symptoms. However, total folate intake from food and supplements in late pregnancy (beta per SD = 0.07, 95% CI: −0.80, 0.93) was not associated with hyperactivity-inattention symptoms	Analysis of red cell folate: child’s sex, mother’s smoking and drinking alcohol during pregnancy, and mother’s educational attainment. Analysis of total folate intake: daily energy, child’s sex
Iodine-ADHD diagnosis or cutoff and ADHD symptoms					
Abel et al. 2017 (Norway) [68]	Prospective cohort; 77,164 children in the MoBa	Iodine intake from foods and supplements assessed by FFQ at GW 22	ADHD identified from Norwegian Patient Registry; ADHD symptom assessed by the ADHD Rating Scale based on mother report at 8 years	Iodine from food was not associated with ADHD diagnosis (*p*-overall = 0.89). Iodine supplement was not associated with ADHD diagnosis, irrespective of food iodine intake. However, higher iodine from food was associated with fewer ADHD symptoms (beta [95% CI] for 25, 50, 75, 100, 125, 200, 225, 250, 300, 350, 400 vs. 160 mcg/day were 0.05 [−0.02, 0.12], 0.06 [0.01, 0.10], 0.06 [0.03, 0.09], 0.05 [0.02, 0.09], 0.03 [0.01, 0.05], −0.01 [-0.03, −0.00], -0.02 [-0.04, 0.01], −0.01 [-0.05, 0.02], −0.01 [-0.07, 0.05], −0.01 [−0.09, 0.08] and −0.00 [−0.12, 0.11], *p*-overall = 0.001) *. Higher iodine supplement was associated with higher ADHD score among women with less than 160 mcg/day of food iodine (beta [95% CI] for 1–200 and >200 mcg/day were 0.06 [0.03, 0.10] and 0.06 [−0.03, 0.16]) *, but not among women with more than 160 mcg/day of food iodine	Sibling clusters, total energy intake, maternal age, BMI, parity, education, smoking in pregnancy, and fiber intake
PUFA and Seafood					
PUFA-ADHD Diagnosis or Cutoff					
Miyake et al. 2018 (Japan) [69]	Prospective cohort; 1199 children in the KOMCHS	Fat intake from food in the past month assessed by FFQ at GW 5–39	Hyperactivity-inattention symptoms assessed by SDQ using mothers’ report at 5 years of age; clinical cutoff was used to define abnormality	Total n-3 PUFA (ORs [95% CI] for quartile 2–4 were 0.82 [0.51, 1.31], 0.75 [0.46, 1.22], and 0.80 [0.49, 1.29], *p*-trend = 0.31), ALA (0.93 [0.59, 1.49], 0.73 [0.44, 1.20], and 0.72 [0.44, 1.18], *p*-trend = 0.13), EPA (0.90 [0.55, 1.46], 0.92 [0.56, 1.49], and 0.97 [0.59, 1.59], *p*-trend = 0.91), and DHA (0.85 [0.52, 1.39], 1.01 [0.62, 1.65], and 1.07 [0.66, 1.73], *p*-trend = 0.66) were not associated with hyperactivity-inattention problem. Total n-6 PUFA (1.05 [0.66, 1.68], 0.75 [0.45, 1.23], and 0.81 [0.49, 1.33], *p*-trend = 0.22), LA (1.03 [0.65, 1.64], 0.67 [0.40, 1.11], and 0.81 [0.49, 1.32], *p*-trend = 0.18), and AA (0.92 [0.56, 1.51], 1.10 [0.69, 1.77], and 0.91 [0.55, 1.50], *p*-trend = 0.92) were not associated with hyperactivity-inattention problem. Total n-3 to n-6 ratio (1.34 [0.83, 2.18], 1.24 [0.75, 2.04], and 0.97 [0.58, 1.63], p-trend = 0.84) was not associated with hyperactivity-inattention problem	Maternal age, gestation at baseline, region of residence at baseline, number of children at baseline, maternal and paternal education, household income, maternal depressive symptoms during pregnancy, maternal alcohol intake during pregnancy, maternal vitamin B complex supplement use during pregnancy, maternal smoking during pregnancy, child’s birth weight, child’s sex, breastfeeding duration, and smoking in the household during the first year of life
PUFA-ADHD symptoms					
Ramakrishnan et al. 2016 (Mexico) [70]	Randomized controlled trial; 797 children in POSGRAD study	Interventions of 400 mg of DHA supplementation or placebo from GW 18–22 to delivery	Hyperactivity-inattention symptoms assessed by K-CPT at 5 years of age. >70th percentile was at clinical risk of suffering from a disorder such as ADHD	DHA supplement of 400 mg/day was not associated with overall K-CPT score >70 (7.2% and 8.1%, *p* = 0.62)	None
Seafood-ADHD diagnosis or cutoff					
Gale et al. 2008 (UK) [71]	Prospective cohort; 219 children	Fish intake in the past 3 months assessed by FFQ at GW 15 and 32	Hyperactivity-inattention symptoms assessed by SDQ using mothers’ report at 9 years of age; clinical cutoff was used to define abnormality	More frequent oily fish intake in early pregnancy (ORs [95% CI] for <1 and ≥1 time/week vs. no intake were 0.30 [0.12, 0.76] and 0.41 [0.15, 1.12]) * and late pregnancy (ORs [95% CI] for <1 and ≥1 time/week vs. no intake were 0.40 [0.16, 0.98] and 0.72 [0.26, 1.98]) * were both associated with lower risk of hyperactivity-inattention problem. Frequency of eating all types of fish was not associated with hyperactivity-inattention problem	Maternal social class, educational qualifications, age, IQ, smoking and drinking in pregnancy, duration of breastfeeding and birthweight
Hibbeln et al. 2007 (UK) [72]	Prospective cohort; 8946 children in ALSPAC	Seafood intake during pregnancy assessed by FFQ at GW 32	Hyperactivity-inattention symptoms assessed by SDQ using mothers’ report at 7 years of age; highest quartile was defined as suboptimal outcome	Seafood intake was not associated with hyperactivity-inattention problem (ORs [95% CI] for none and 1–340 g/week vs. ≥340 g/week were 1.13 [0.84, 1.53] and 0.91 [0.73, 1.12], *p*-trend = 0.66)	Maternal education, housing, crowding at home, life events, partner, maternal age, maternal smoking in pregnancy, maternal alcohol use in pregnancy, parity, breastfeeding, gender, ethnic origin, birthweight, preterm delivery, 12 non-fish food groups
Caffeine, coffee and tea					
Caffeine-ADHD diagnosis or cutoff					
Miyake et al. 2018 (Japan) [73]	Prospective cohort; 1199 children in the KOMCHS	Caffeine intake from food in the past month assessed by FFQ at GW 5–39	Hyperactivity-inattention symptoms assessed by SDQ using mothers’ report at 5 years of age; clinical cutoff was used to define abnormality	Caffeine was not associated with hyperactivity-inattention problem (ORs [95% CI] for quartile 2–4 were 1.04 [0.64, 1.68], 0.99 [0.61, 1.62], and 0.84 [0.51, 1.38], *p*-trend = 0.49)	Maternal age, gestation at baseline, region of residence at baseline, number of children at baseline, maternal and paternal education, household income, maternal depressive symptoms during pregnancy, maternal alcohol intake during pregnancy, maternal vitamin B complex supplement use during pregnancy, maternal smoking during pregnancy, child’s birth weight, child’s sex, breastfeeding duration, and smoking in the household during the first year of life
Del-Ponte et al. 2016 (Brazil) [74]	Prospective cohort; 3485 children	Caffeine intake during each trimester assessed in interviews after delivery	ADHD assessed by DAWBA based on mother’s report at 11 years of age; clinical cutoff was used to define abnormality	Caffeine in the entire pregnancy was not associated with ADHD risk (ORs [95% CI] for 100–299 and ≥300 vs. <100 mg/day were 1.12 [0.68 to 1.84] and 0.90 [0.51 to 1.59]). Similar results were found in each of the three trimesters	Maternal mood symptoms during pregnancy, National Economic Index, paternal education level and maternal conjugal situation
Loomans et al. 2012 (The Netherlands) [75]	Prospective cohort; 3439 children in the ABCD study	Caffeine intake from coffee, tea and cola in the past week assessed by questionnaire at GW 16	Hyperactivity-inattention symptoms assessed by SDQ based on mothers’ report at 5–6 years of age; clinical cutoff was used to define abnormality	Caffeine was not associated with hyperactivity-inattention problem (ORs [95% CI] for 86–255, 256–425, and >425 vs. 0–85 mg/day were 0.94 [0.68, 1.31], 0.87 [0.57, 1.33], and 1.08 [0.55, 2.12])	Maternal age, ethnicity, maternal education, maternal anxiety, cohabitant status, smoking, alcohol, child’s gender, family size
Coffee and tea-ADHD diagnosis or cutoff					
Hvolgaard Mikkelsen et al. 2017 (Denmark) [76]	Prospective cohort; 47,491 children in the DNBC	Current coffee and tea intake assessed by interviews at GW 15 and 30	Hyperactivity-inattention symptoms assessed by SDQ based on children, parents and teachers’ report at 11 years of age; computerized algorithms were used to identified ADHD	Higher coffee intake in the first trimester was associated with higher risk of hyperactivity-inattention problem (ORs [95% CI] for 1–3, 4–7 and ≥8 cups/day vs. no intake were 0.97 [0.88, 1.08], 1.09 [0.93, 1.27] and 1.47 [1.18, 1.83], *p*-trend = 0.03) *. In the third trimester, it was not associated (ORs [95% CI] for 1–3, 4–7 and ≥8 cups/day vs. no intake were 0.94 [0.85, 1.04], 0.96 [0.83, 1.13] and 1.21 [0.95, 1.55], *p*-trend = 0.88). Tea intake in the first trimester was not associated with hyperactivity-inattention problem (ORs [95% CI] for 1–3, 4–7 and ≥8 cups/day vs. no intake were 0.93 [0.85, 1.03], 0.97 [0.85, 1.12] and 1.21 [0.98, 1.49], *p*-trend = 0.57). In the third trimester, higher tea intake was associated with lower risk of hyperactivity-inattention problem (ORs [95% CI] for 1–3, 4–7 and ≥8 cups/day vs. no intake were 0.90 [0.81, 1.01], 0.79 [0.66, 0.94] and 0.84 [0.63, 1.11], *p*-trend = 0.01) *	Sex, birth year, smoking, socioeconomic status, maternal age, parity, maternal BMI, and mutually coffee or tea
Linnet et al. 2009 (Denmark) [77]	Prospective cohort; 24,068 children in the Aarhus Birth Cohort	Coffee intake during pregnancy assessed by a questionnaire prior to GW 16	Hyperkinetic disorder and ADHD recorded in Danish Psychiatric Central Register; children were 3–12 years of age	Coffee was not associated with ADHD risk (RRs [95% CI] for 1–3, 4–9 and ≥10 cups of coffee were 0.9 [0.5, 1.6], 1.3 [0.7, 2.3] and 2.3 [0.9, 5.9])	Smoking, alcohol, maternal age, gender of the child, parental years of schooling after basic school, employment status, cohabitant status and parental and sibling’s psychiatric hospitalizations or contacts as outpatients
Dietary patterns					
Dietary patterns-ADHD diagnosis or cutoff					
Galera et al. 2018 (France) [78]	Prospective cohort; 1242 children in the EDEN mother-child cohort	Dietary patterns in the third trimester assessed by FFQ after delivery and derived using principle component analysis	Hyperactivity-inattention symptoms assessed by SDQ based on mother’s report at 3, 5, and 8 years of age; clinical cutoff was used to define abnormality; longitudinal trajectories were derived based on mixture models	Lower scores of healthy dietary pattern (OR [95% CI] for quartile 1 vs. the rest was 1.61 [1.09, 2.37]) * and higher scores of Western dietary pattern (OR for quartile 4 vs. the rest was 1.67 [1.13, 2.47]) * were both associated with higher risk of high hyperactivity-inattention trajectory	Centre, child gender, maternal age, prepregnancy BMI, maternal smoking, maternal alcohol-drinking, gestational diabetes, multiparity, gestational length, birth weight, breastfeeding, prenatal maternal depressive symptoms, prenatal maternal anxiety, postnatal maternal depressive symptoms, parental separation, family income, maternal education, child dietary patterns at age 2, and mutual adjusted for healthy and Western dietary patterns
Dietary patterns-ADHD symptoms					
Rijlaarsdam et al. 2017 (UK) [56]	Prospective cohort; 83 youths with early-onset persistent conduct problems and 81 youths with low conduct problem in ALSPAC	Dietary patterns during pregnancy assessed by FFQ at GW 32 and derived using confirmatory factor analysis	ADHD symptoms assessed by DAWBA based on parent reports at 7 years of age	Unhealthy dietary pattern was indirectly associated with more ADHD symptoms through *IGF2* DNA methylation at birth among youths with early-onset persistent conduct problems (beta per SD = 0.069, 95% CI: 0.003, 0.206) *, but not among youths with low conduct problem (beta per SD = −0.015, 95% CI: −0.086, 0.019)	Cumulative risk index during pregnancy and in childhood, including life events, contextual risks, parental risks, interpersonal risks, direct victimization

* Statistically significant findings (*p*-value < 0.05). Abbreviates of research studies: ABCD—Amsterdam Born Children and their Development; ALSPAC—Avon Longitudinal Study of Parents and Children; DNBC—Danish National Birth Cohort; EDEN—Étude des Déterminants pré et postnatals du développement et de la santé de l’ENfant; KOMCHS—Kyushu Okinawa Maternal and Child Health Study; MoBa—Norwegian Mother and Child Cohort Study; POSGRAD—Prenatal Omega-3 Fatty Acid Supplementation and Child Growth and Development. Other abbreviations: ADHD—attention—deficit/hyperactivity disorder; BMI—body mass index; CI—confidence interval; DAWBA—Development and Well—Being Assessment; DHA—docosahexaenoic acid; FFQ—food frequency questionnaire; GW—gestational week; HR—hazard ratio; K-CPT—Conner’s Kiddie Continuous Performance Test; OR—odds ratio; RR—relative risk; SD—standard deviation; SDQ—Strengths and Difficulties Questionnaire.

## Supplementary Materials

The following are available online at https://www.mdpi.com/2072-6643/11/7/1628/s1, Table S1: Search strategy and history in PubMed and Embase; Figure S1: Adjusted relative risk (RR) of offspring risk of autism spectrum disorder associated with maternal intake of multivitamin or prenatal vitamin and supplement with folic acid only formulation during pregnancy.
