# Supplementary material for: Preconception and Prenatal Nutrition and Neurodevelopmental Disorders: A Systematic Review and Meta-Analysis
Source: Nutrients. 2019 Jul 17;11(7):1628. doi: 10.3390/nu11071628 (PMC6682900; doi:10.3390/nu11071628)
Supplement: Supplementary file 1 [file nutrients-11-01628-s001.pdf]

Supplementary Materials

Table S1. Search strategy and history in PubMed and Embase

#	Query	Records
**PubMed Search**		
#1	Search neurodevelopmental disorders[mh] OR "neurodevelopmental disorde*"[tiab] OR developmental disabilities[mh] OR "developmental disabilit*"[tiab] OR “developmental delay”[tiab] OR autism spectrum disorder[mh] OR autistic disorder[mh] OR autism[tiab] OR autistic[tiab] OR ASD[tiab] OR “Asperger Syndrome”[mh] OR “Asperger”[tiab] OR child development disorders, pervasive[mh] OR "pervasive developmental disorde*"[tiab] OR intellectual disability[mh] OR "intellectual disability"[tiab] OR "mental retardation"[tiab] OR "general learning disability"[tiab] OR “intellectual developmental disorder”[tiab] OR “intellectual impairment”[tiab] OR “mental impairment”[tiab] OR “mental handicap”[tiab] OR “intellectual deficiency”[tiab] OR “mental deficiency”[tiab] OR attention deficit disorder with hyperactivity[mh] OR adhd[tiab] OR "attention deficit"[tiab] OR "attention-deficit"[tiab] OR inattention[tiab] OR inattentive[tiab] OR hyperactivity[tiab] OR hyperactive[tiab] OR hyperkinetic[tiab]	281,058
#2	Search nutrients[mh] OR nutrien*[tiab] OR micronutrients[mh] OR micronutrien*[tiab] OR "trace elemen*"[tiab] OR vitamins[mh] OR vitami*[tiab] OR macronutrien*[tiab] OR dietary proteins[mh] OR proteins[tiab] OR protein[tiab] OR dietary carbohydrates[mh] OR carbohydrat*[tiab] OR dietary fiber[mh] OR fiber[tiab] OR polysaccharides[mh] OR polysaccharid*[tiab] OR vitamin a[mh] OR "vitamin a"[tiab] OR folic acid[mh] OR folate[tiab] OR "folic acid"[tiab] OR Vitamin B 12[mh] OR "vitamin B12"[tiab] OR ascorbic acid[mh] OR "vitamin C"[tiab] OR vitamin d[mh] OR "vitamin D"[tiab] OR calcium, dietary[mh] OR calcium[mh] OR calcium[tiab] OR iron, dietary[mh] OR iron[tiab] OR ((magnesium[mh] OR magnesium deficiency[mh] OR magnesium[tiab]) NOT (Magnesium Sulfate[mh] OR magnesium[tiab])) OR iodine[mh] OR iodine[tiab] OR choline[mh] OR choline[tiab] OR phosphorus, dietary[mh] OR "phosphorus"[tiab] OR dietary fats[mh] OR "dietary fats"[tiab] OR "dietary fat"[tiab] OR lipids[mh] OR lipids[tiab] OR lipid[tiab] OR fats[mh] OR fat[tiab] OR fats[tiab] OR "saturated fats"[tiab] OR "saturated fat"[tiab] OR fatty acids[mh] OR "fatty acids"[tiab] OR "fatty acid"[tiab] OR fatty acids, unsaturated[mh] OR "unsaturated fatty acids"[tiab] OR "unsaturated fatty acid"[tiab] OR fatty acids, Omega-3[mh] OR "omega-3 fatty acids"[tiab] OR docosahexaenoic acids[mh] OR "docosahexaenoic acid"[tiab] OR fatty acids, monounsaturated[mh] OR "monounsaturated fatty acids"[tiab] OR food[tiab] OR foods[tiab] OR edible grain[mh] OR grains[tiab] OR red meat[mh] OR "red meat"[tiab] OR fish products[mh] OR seafood[mh] OR fish[tiab] OR seafood[tiab] OR seafoods[tiab] OR fruit[mh] OR fruit[tiab] OR fruits[tiab] OR vegetables[mh] OR vegetabl*[tiab] OR eggs[mh] OR eggs[tiab] OR potatoes[tiab] OR potato[tiab] OR dairy products[mh] OR dairy[tiab] OR caffeine[mh] OR caffein*[tiab] OR	6,117,149

	coffee[tiab] OR nuts[mh] OR nuts[tiab] OR "processed food"[tiab] OR "processed foods"[tiab] OR (("sugar-sweetened beverages"[tiab] OR "artificially sweetened beverages"[tiab] OR ((sweetening agents[mh] OR "artificial sweeteners"[tiab] OR "artificial sweeteners"[tiab] OR "non-nutritive sweeteners"[tiab] OR "non-nutritive sweetener"[tiab]) AND (beverages[mh] OR beverage[tiab] OR drinks[tiab] OR soda[tiab] OR sodas[tiab]))) NOT (infant food[mh] OR foods, specialized[mh] OR crops, agricultural[mh] OR "animal feed"[tiab])) OR diet[mh] OR diet[tiab] OR diets[tiab] OR "dietary pattern"[tiab] OR "dietary patterns"[tiab] OR "dietary quality"[tiab] OR "eating pattern"[tiab] OR "food pattern"[tiab] OR "eating habit"[tiab] OR "dietary habit"[tiab] OR "food habit"[tiab] OR "dietary profile"[tiab] OR "food profile"[tiab] OR "diet profile"[tiab] OR "eating profile"[tiab] OR "eating style"[tiab] OR Diet, high-fat[mh] OR "fat intake"[tiab] OR "high-fat diet"[tiab] OR diet, western[mh] OR "western diet"[tiab] OR diet, mediterranean[mh] OR "mediterranean diet"[tiab] OR "dash diet"[tiab] OR "dietary approaches to stop hypertension"[tiab] OR (DASH[tiab] AND diet[tiab]) OR "healthy eating index"[tiab] OR diet, vegetarian[mh] OR "vegetarian diet"[tiab] OR vegan[tiab] OR vegetarian[tiab] OR diet, carbohydrate-restricted[mh] OR diet, fat-restricted[mh] OR diet, protein-restricted[mh] OR diet, sodium-restricted[mh] OR "nutrient restrict*"[tiab] OR diet, carbohydrate loading[mh] OR "glycemic load"[tiab] OR high glycemic[tiab] OR "carbohydrate load"[tiab] OR "low carbohydrate diet"[tiab] OR "prudent diet"[tiab] OR nordiet[tiab] OR omniheart[tiab] OR "Optimal Macronutrient Intake Trial to Prevent Heart Disease"[tiab] OR adventist*[tiab] OR ((Okinawa* OR "Ethnic Groups"[Mesh] OR "plant based" OR Nordic[tiab] OR "heart healthy"[tiab] OR indo-mediterranean) AND (diet[mh] OR diet[tiab] OR diets[tiab] OR food[mh]))	
#3	Search Prenatal Exposure Delayed Effects[mh] OR (("intra-uter*"[tiab] OR "intrauter*"[tiab] OR "in-uter*"[tiab] OR fetal[tiab] OR foetal[tiab] OR prenatal[tiab] OR developmental[tiab]) AND (origins[tiab] OR programming[tiab])) OR "metabolic programming"[tiab] OR (("intra-uter*"[tiab] OR "intrauter*"[tiab] OR "in-uter*"[tiab] OR fetal[tiab] OR foetal[tiab] OR prenatal[tiab] OR perinatal[tiab] OR periconcept*[tiab] OR intergeneratio*[tiab] OR transgeneratio*[tiab] OR maternal[tiab]) AND (effects[tiab] OR exposure[tiab] OR exposures[tiab] OR nutrition[tiab] OR diet[mh] OR diet[tiab] OR environment[tiab] OR environments[tiab])) OR pregnancy[mh] OR pregnan*[tiab]	1,022,196
#4	cohort studies[mesh] OR "case-control studies"[mesh] OR "comparative study"[pt] OR "risk factors"[mesh] OR "cohort"[tw] OR "compared"[tw] OR "groups"[tw] OR "case control"[tw] OR "multivariate"[tw] OR "randomized controlled trial"[pt] OR "controlled clinical trial"[pt] OR "clinical trials as topic"[mesh] OR "random allocation"[mesh] OR "double-blind method"[mesh] OR "single-blind method"[mesh] OR "clinical trial"[pt] OR "research design"[mesh:noexp] OR "comparative study"[pt] OR "evaluation studies"[pt] OR "follow-up studies"[mesh] OR "prospective studies"[mesh]	10,348,850

	OR "cross-over studies"[mesh] OR "clinical trial"[tw] OR ((singl*[tw] OR doubl*[tw] OR trebl*[tw]) AND (mask*[tw] OR blind*[tw])) OR placebo*[tw] OR random*[tw] OR "control"[tw] OR "controls"[tw] OR prospectiv*[tw] OR volunteer*[tw]	
#5	Search animals[mesh:noexp]	6,376,150
**#6**	**Search ((#1 AND #2 AND #3 AND #4) NOT #5)**	**1,393**
	**Embase Search**	
#1	'neurodevelopmental delay'/exp OR 'neurodevelopmental disorder'/exp OR 'developmental disorder'/exp OR 'developmental disability' OR 'developmental delay':ti,ab OR 'autism'/exp/mj OR 'autistic disorder':ti,ab OR 'autism':ti,ab OR 'autistic':ti,ab OR 'asd':ti,ab OR 'child pervasive developmental disorders':ti,ab OR (pervasive AND developmental AND disord*:ti,ab) OR 'asperger syndrome'/exp OR 'asperger':ti,ab OR 'intellectual impairment':de OR 'intellectual impairment':ti,ab OR (intellectual AND disability*:ti,ab) OR (development* NEAR/2 (delay* OR disabilit* OR disorder*)) OR 'mental deficiency' OR (mental* NEAR/2 (retard* OR disability* OR disorder*)) OR (intellect* NEAR/2 (disabilit* OR delay* OR disorder*)) OR 'general learning disabilit':ti,ab OR 'intellectual developmental disorder' OR 'intellectual impairment' OR 'mental impairment' OR 'mental handicap' OR 'cognitive delay':ti,ab OR 'intellectual deficit' OR 'mental deficit' OR 'attention deficit disorder'/exp OR 'attention deficit disorder with hyperactivity':ti,ab OR adhd:ti,ab OR 'attention deficit':ti,ab OR 'attention-deficit':ti,ab OR 'inattention' OR 'inattentive' OR 'hyperactivity' OR 'hyperactive' OR 'hyperkinetic'	427,106
#2	('maternal nutrition'/mj OR (maternal:ab,ti AND nutrient*:ab,ti) OR 'nutrients'/mj OR nutrien*:ab,ti OR (trace:an,ti AND elemen*:an,ti) OR 'vitamin'/mj OR vitami*:ab,ti OR macronutrien*:ab,ti OR 'protein intake'/mj OR 'proteins':ab,ti OR 'protein':ab,ti OR 'carbohydrate diet'/mj OR carbohydrat*:ab,ti OR 'dietary fiber'/mj OR 'fiber':ab,ti OR 'polysaccharide'/mj OR polysaccharide*:ab,ti OR 'retinol'/mj OR 'vitamin a':ab,ti OR 'folic acid'/mj OR 'folate':ab,ti OR 'folic acid':ab,ti OR 'cyanocobalamin'/mj OR 'vitamin b12':ab,ti OR 'ascorbic acid'/mj OR 'vitamin c':ab,ti OR 'vitamin d'/mj OR 'vitamin d':ab,ti OR 'calcium intake'/mj OR 'calcium'/mj OR 'calcium':ab,ti OR 'iron intake'/mj OR 'iron':ab,ti OR (('magnesium'/mj OR 'magnesium deficiency'/mj OR 'magnesium':ab,ti) NOT ('magnesium sulfate'/mj OR 'magnesium':ab,ti)) OR 'iodine'/mj OR 'iodine':ab,ti OR 'choline'/mj OR 'choline':ab,ti OR 'phosphorus intake'/mj OR 'phosphorus':ab,ti OR 'edible oil'/mj OR 'fat intake'/mj OR 'dietary fats':ab,ti OR 'dietary fat':ab,ti OR 'lipid'/mj OR 'lipids':ab,ti OR 'lipid':ab,ti OR 'fats'/mj OR 'fat':ab,ti OR 'fats':ab,ti OR 'saturated fats':ab,ti OR 'saturated fat':ab,ti OR 'fatty acids'/mj OR 'fatty acids':ab,ti OR 'fatty acid':ab,ti OR 'unsaturated fatty acid'/mj OR 'unsaturated fatty acids':ab,ti OR 'unsaturated fatty acid':ab,ti OR 'omega 3 fatty acid'/mj OR 'omega-3 fatty acids':ab,ti OR 'docosahexaenoic acid'/mj OR 'docosahexaenoic acid':ab,ti OR 'monounsaturated fatty acid'/mj OR 'monounsaturated fatty acids':ab,ti OR 'food':ab,ti OR 'foods':ab,ti OR 'food grain'/mj OR 'grains':ab,ti OR 'meat'/mj OR 'red meat'/mj OR 'red	6,220,414

	meat':ab,ti OR 'fish products'/mj OR 'fish meat'/mj OR 'sea food'/mj OR 'fish':ab,ti OR 'seafood':ab,ti OR 'seafoods':ab,ti OR 'fruit'/mj OR 'fruit':ab,ti OR 'fruits':ab,ti OR 'vegetables'/mj OR vegetabl*:ab,ti OR 'egg'/mj OR 'eggs':ab,ti OR 'potatoes':ab,ti OR 'potato':ab,ti OR 'dairy product'/mj OR 'dairy':ab,ti OR 'caffeine'/mj OR caffein*:ab,ti OR 'coffee':ab,ti OR 'nut'/mj OR 'nuts':ab,ti OR 'processed food':ab,ti OR 'processed foods':ab,ti OR (('sugar-sweetened beverages':ab,ti OR 'artificially sweetened beverages':ab,ti OR 'sweetening agents'/mj OR 'artificial sweeteners':ab,ti OR 'non-nutritive sweeteners':ab,ti OR 'non-nutritive sweetener':ab,ti) AND ('beverage'/mj OR 'beverage':ab,ti OR 'drinks':ab,ti OR 'soda':ab,ti OR 'sodas':ab,ti))) NOT ('infant food'/mj OR 'crops'/mj OR 'animal food':ab,ti) OR 'diet'/mj OR 'diet':ab,ti OR 'diets':ab,ti OR 'dietary pattern':ab,ti OR 'dietary patterns':ab,ti OR 'dietary quality':ab,ti OR 'eating pattern':ab,ti OR 'food pattern':ab,ti OR 'eating habit':ab,ti OR 'dietary habit':ab,ti OR 'food habit':ab,ti OR 'dietary profile':ab,ti OR 'food profile':ab,ti OR 'diet profile':ab,ti OR 'eating profile':ab,ti OR 'eating style':ab,ti OR 'high fat/sucrose diet' OR 'high fat/fructose diet' OR 'fat intake':ab,ti OR 'high-fat diet':ab,ti OR 'western diet'/mj OR 'western diet':ab,ti OR 'mediterranean diet'/mj OR 'mediterranean diet':ab,ti OR 'dash diet':ab,ti OR 'dietary approaches to stop hypertension':ab,ti OR ('dash':ab,ti AND 'diet':ab,ti) OR 'healthy eating index':ab,ti OR 'vegetarian diet'/mj OR 'vegetarian diet':ab,ti OR 'vegan':ab,ti OR 'vegetarian':ab,ti OR 'low carbohydrate diet'/mj OR 'fat restricted diet':ab,ti OR 'protein restricted diet':ab,ti OR 'sodium restricted diet':ab,ti OR (nutrient:ab,ti AND restrict*:ab,ti) OR 'carbohydrate loading diet'/mj OR 'glycemic load':ab,ti OR 'high glycemic':ab,ti OR 'carbohydrate load':ab,ti OR 'low carbohydrate diet':ab,ti OR 'prudent diet':ab,ti OR 'nordiet':ab,ti OR 'omniheart':ab,ti OR 'optimal macronutrient intake trial to prevent heart disease':ab,ti OR adventist*:ab,ti OR ((okinawa* OR 'ethnic groups'/mj OR 'plant based' OR 'nordic':ab,ti OR 'heart healthy':ab,ti OR 'indo-mediterranean':ab,ti) AND ('diet'/mj OR 'diet':ab,ti OR 'diets':ab,ti OR 'food'/mj))	
#3	'prenatal exposure'/mj/exp OR (('intra uter*':ab,ti OR intrauter*:ab,ti OR 'in uter*':ab,ti OR 'fetal':ab,ti OR 'foetal':ab,ti OR 'prenatal':ab,ti OR 'developmental':ab,ti) AND ('origins':ab,ti OR 'programming':ab,ti)) OR 'metabolic programming':ab,ti OR (('intra uter*':ab,ti OR intrauter*:ab,ti OR 'in uter*':ab,ti OR 'fetal':ab,ti OR 'foetal':ab,ti OR 'prenatal':ab,ti OR 'perinatal':ab,ti OR periconcept*:ab,ti OR intergeneratio*:ab,ti OR transgeneratio*:ab,ti OR 'maternal':ab,ti) AND ('effects':ab,ti OR 'exposure':ab,ti OR 'exposures':ab,ti OR 'nutrition':ab,ti OR 'diet'/exp/mj OR 'diet':ab,ti OR 'environment':ab,ti OR 'environments':ab,ti)) OR 'pregnancy'/exp/mj OR pregnan*:ab,ti	814,692
#4	'human'/de AND ('case report'/de OR 'clinical article'/de OR 'clinical trial'/de OR 'cohort analysis'/de OR 'comparative study'/de OR 'controlled clinical trial'/de OR 'controlled study'/de OR 'cross-sectional study'/de OR 'human'/de OR 'human cell'/de OR 'human tissue'/de OR 'major clinical study'/de OR 'normal human'/de OR 'prospective study'/de OR 'randomized controlled	12,812,066

	trial'/de OR 'retrospective study'/de) NOT ('review'/de OR 'meta-analysis'/de OR 'nohuman' OR 'animal experiement') AND 'article'/it	
**#5**	**#1 AND #2 AND #3 AND #4**	**1,101**

Figure S1. Adjusted relative risk (RR) of offspring risk of autism spectrum disorder associated with maternal intake of multivitamin or prenatal vitamin (top) and supplement with folic acid only formulation (bottom) during pregnancy.

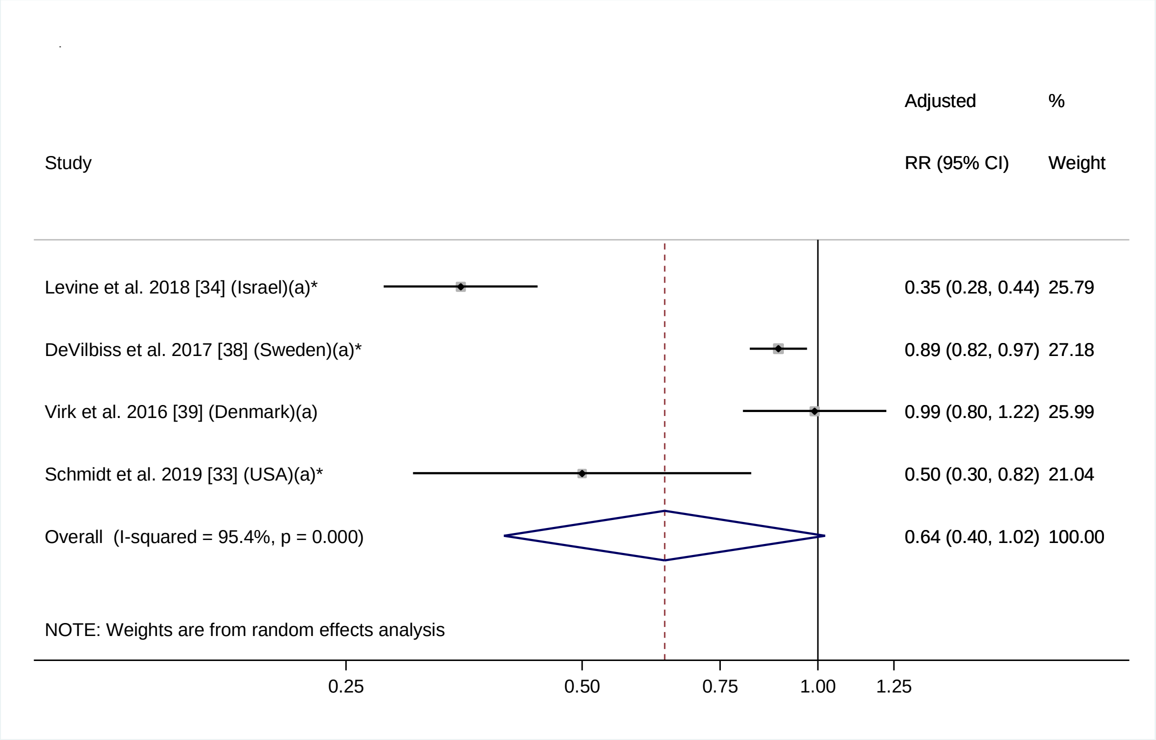

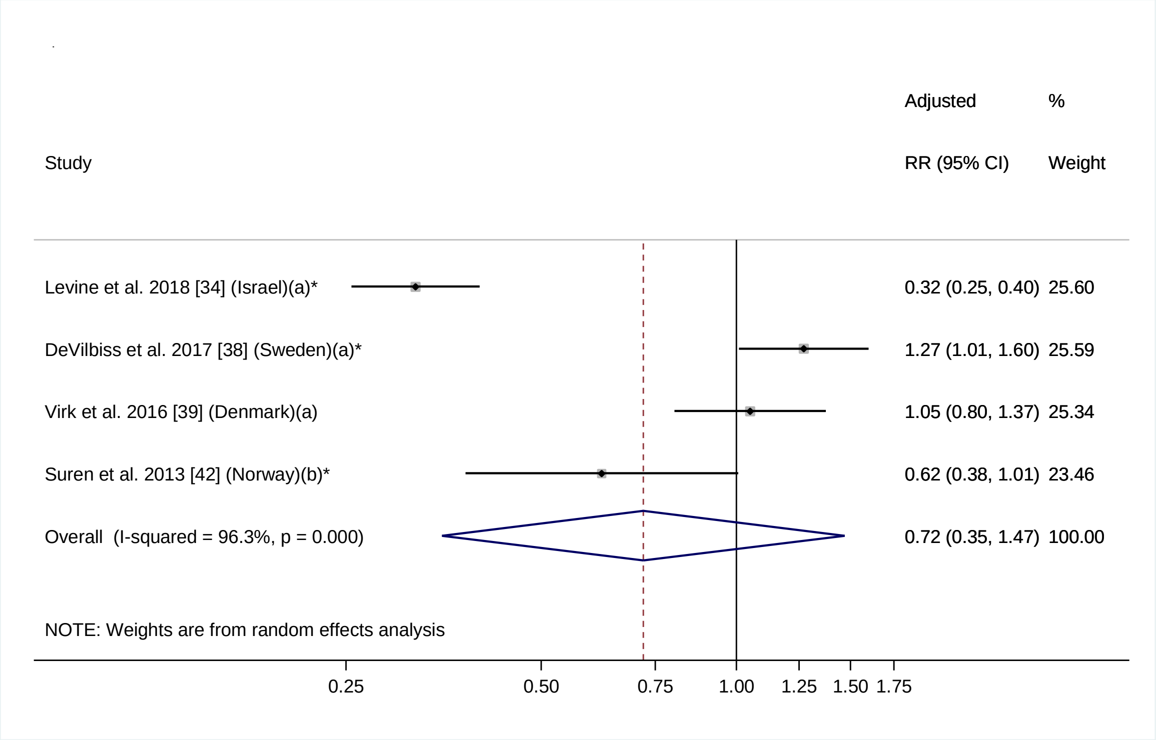

The overall effect size was estimated using random effects models weighted by inverse variance of each study. (a) During pregnancy; (b) Before and during pregnancy
